# Transcriptomic-metabolomic reprogramming in EGFR-mutant NSCLC early adaptive drug escape linking TGFβ2-bioenergetics-mitochondrial priming

**DOI:** 10.18632/oncotarget.13307

**Published:** 2016-11-11

**Authors:** Praveena S. Thiagarajan, Xiaoliang Wu, Wei Zhang, Ivy Shi, Rakesh Bagai, Patrick Leahy, Yan Feng, Martina Veigl, Daniel Lindner, David Danielpour, Lihong Yin, Rafael Rosell, Trever G. Bivona, Zhenfeng Zhang, Patrick C. Ma

**Affiliations:** ^1^ Translational Hematology and Oncology Research, Taussig Cancer Institute, Cleveland Clinic, Cleveland, OH, USA; ^2^ Lerner Research Institute, Cleveland Clinic, Cleveland, OH, USA; ^3^ Sun Yat-sen University Cancer Center, State Key Laboratory of Oncology in South China & Collaborative Innovation Center for Cancer Medicine, Guangzhou, China; ^4^ Case Comprehensive Cancer Center, Cleveland, OH, USA; ^5^ Department of Pharmacology, and Department of Biochemistry, Case Western Reserve University, Cleveland, OH, USA; ^6^ Catalan Institute of Oncology, Badalona, Spain; ^7^ Spanish Lung Cancer Group, Badalona, Spain; ^8^ Department of Medicine, Division of Hematology/Oncology, Helen Diller Comprehensive Cancer Center, University of California San Francisco, San Francisco, CA, USA; ^9^ Sara Crile Allen and James Frederick Allen Comprehensive Lung Cancer Program, Eminent Scholar in Lung Cancer Research, WVU Cancer Institute, West Virginia University, Morgantown, WV, USA

**Keywords:** EGFR, inhibitor, drug escape, resistance, lung cancer

## Abstract

The impact of *EGFR*-mutant NSCLC precision therapy is limited by acquired resistance despite initial excellent response. Classic studies of *EGFR*-mutant clinical resistance to precision therapy were based on tumor rebiopsies late during clinical tumor progression on therapy. Here, we characterized a novel non-mutational early adaptive drug-escape in *EGFR*-mutant lung tumor cells only days after therapy initiation, that is MET-independent. The drug-escape cell states were analyzed by integrated transcriptomic and metabolomics profiling uncovering a central role for autocrine TGFβ2 in mediating cellular plasticity through profound cellular adaptive **Omics** reprogramming, with common mechanistic link to prosurvival mitochondrial priming. Cells undergoing early adaptive drug escape are in proliferative-metabolic quiescent, with enhanced EMT-ness and stem cell signaling, exhibiting global bioenergetics suppression including reverse Warburg, and are susceptible to glutamine deprivation and TGFβ2 inhibition. Our study further supports a preemptive therapeutic targeting of bioenergetics and mitochondrial priming to impact early drug-escape emergence using EGFR precision inhibitor combined with broad BH3-mimetic to interrupt BCL-2/BCL-xL together, but not BCL-2 alone.

## INTRODUCTION

EGFR tyrosine kinase inhibitors (TKIs), such as erlotinib and gefitinib are approved precision therapies for advanced non-small cell lung cancer (NSCLC) treatment in patients with drug-sensitizing *EGFR*-mutant tumors [1–3]. As a result of “oncogene-addiction”, EGFR TKIs often yields tumor response that can be rapid and remarkable [4]. Nonetheless, acquired drug resistance invariably develops later in the course of therapy despite initial response, leading to ultimate therapeutic failure and patient demise even in prior near-complete/complete responders. Prior paired rebiopsy studies typically emphasized on the late phase of therapy, when clinically evident tumor resistant progression became detectable [5–11]. Diverse mechanisms of EGFR-TKI acquired late resistance have been described, e.g. T790M-*EGFR* [8, 12], *MET*/*HGF* activation, *HER2* amplification, *PIK3CA* mutation, small cell histologic transformation [5–9] and epithelial-mesenchymal transition (EMT) mediated by *AXL*/*GAS6* upregulation [10]. Emphasizing on early changes of EGFR/MET inhibitors on drug-sensitive lung tumor cells [13], we have recently identified and characterized the early onset adaptive precision drug escape that emerged from the treated drug-sensitive parental cell population, after TKI exposure for as short as merely 6-9 days. These cells in drug escape exhibited ~100-fold higher drug-resistance phenotype, and a MET-independent but enhanced dependence on the intrinsic mitochondrial prosurvival signaling cascade [13]. Our study also showed that while undergoing drug escape, these cells had profoundly inhibited adaptive state of proliferation, cell motility and migration (http://cancerres.aacrjournals.org/content/71/13/4494/suppl/DC1).

In the present study, we conducted transcriptome- and metabolome-wide profiling to comprehensively characterize the cellular state of the early adaptive drug escape in *EGFR-*mutant NSCLC under EGFR-TKI. In recent years, cancer metabolism has emerged as a key concept capable of providing deeper understanding of human malignancies and is now known to integrate closely with the oncogenic kinase signaling [14]. Altered metabolism is now viewed as a core Hallmark of Cancer [15], rather than merely an indirect response to cell proliferative and survival signals. We report here that transforming growth factor-beta 2 (TGFβ2) autocrine upregulation plays a central role in the early adaptive omics reprogramming and drug escape in *EGFR-*mutant NSCLC. Our results showed that within the drug escape process, there is a link between TGFβ2 and adaptive global cellular reprogramming involving bioenergetics-mitochondrial BCL-2/BCL-xL cascades to promote a prosurvival cell state embedded in the proliferative-metabolic quiescence. Furthermore, we demonstrated now that the early adaptive EGFR-TKI drug-resistant cells are sensitive to TGFβ2 inhibition and glutamine withdrawal.

## RESULTS

### Genome-wide expression profiling of *EGFR*-mutant NSCLC early adaptive drug escape against EGFR Inhibitor

**Figure 1:**
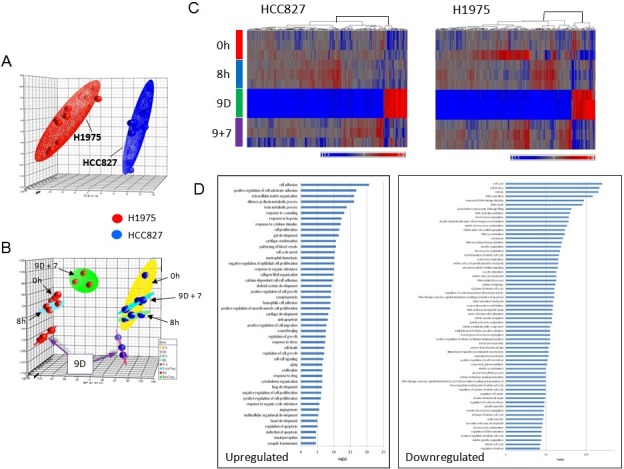
Principal component analysis (PCA) of precision therapy of *EGFR*-mutant drug-sensitive lung adenocarcinoma cells **1A.** Segregation of HCC827 (deletion exon 19-*EGFR*) and H1975 (T790M/L858R-*EGFR*) cells in the two separate preclinical *in vitro* model systems. Transcriptome-wide profiling of early adaptive drug-escape in *EGFR*-mutant lung adenocarcinoma was analyzed using PCA. HCC827 cells were treated with erlotinib at 1 μM and H1975 cells were treated with CL-387,785 at 1 μM for 9 days followed by TKI washout for another 7 days in biologic triplicates. **1B.** PCA of the gene expression data from the microarray gene expression profiling study as above in **1C.** Transcriptomic profiling of early adaptive drug-escape against EGFR-TKI in *(a)* HCC827 and *(b)* H1975 lung adenocarcinoma cells - Clustering heat map analysis. **1D.** Pathway analysis from the Affymetrix gene expression microarray studies in early adaptive drug-escape against EGFR-TKI in *EGFR*-mutant lung adenocarcinoma.

We have recently reported a MET-independent early onset adaptive drug escape among the parental drug-sensitive *EGFR*-mutant lung adenocarcinoma cells (HCC827 and PC-9 to erlotinib; H1975 to CL-387,785)[13]. Here, we extended our studies to perform genome expression profiling analysis of the *EGFR*-mutant NSCLC in early adaptive escape against the EGFR inhibitor. We used the HCC827 cells (deletion exon 19-*EGFR:* p.Glu746_Ala750del) inhibited by erlotinib (reversible EGFR TKI) and H1975 cells (T790M/L858R-*EGFR*) inhibited by CL-387,785 (irreversible EGFR TKI) as *in vitro* preclinical model. Principal component analysis (PCA) revealed that the two cell line systems segregated well in their gene expression signature profiles (Figure 1A-1B). These results validated the model systems and the treatment conditions adopted in this study. Next, we performed BAMarray analysis (incorporating Bayesian Analysis of Microarray data) of the gene expression microarray datasets (Figure 1C). We found that there were dramatic transcriptome expression landscape changes during the 9 days of EGFR TKI treatment, resulting in a unique and highly homogeneous altered gene expression signature pattern in the early onset adaptive drug-evading cells at day 9 TKI treatment. Importantly, this reprogramming of transcriptomic signature is evident in both cell line models using HCC827 (Figure 1C, left) and H1975 cells (Figure 1C, right) under the corresponding TKI treatment with erlotinib and CL-387,785 respectively.

Common pathway analysis of the significantly upregulated and downregulated genes in both cell line models was then conducted to identify key signaling pathways that are involved in mediating the early adaptive drug escape (Figure 1D, table S1, S2). We identified that upregulated TGFβ2 potentially played a central role and was significantly involved in a multitude of signaling pathways in the cells undergoing early adaptive drug escape (table S1). Of note, among the top significantly upregulated pathway genes within the early adaptive drug escaping cells include those regulating cell adhesion, cell proliferation, multicellular organismal development, extracellular matrix organization, and response to hypoxia, among others. On the other hand, pathway genes that were significantly downregulated in these cells converged into pathways regulating cell cycle (arrest and progression), mitosis, cell proliferation, DNA repair, nucleosome assembly, chromatin organization and histone modification (table S2). Of particular interest, several key metabolic enzyme genes involved in glucose metabolism: glucose-6-phosphate isomerase (*GPI*), phosphoglycerate kinase 1 (*PGK1*) and enolase 2 (*ENO2*), were found to be downregulated.

### Autocrine TGFβ2 in the early adaptive EGFR-TKI drug escape and reprogramming of EMT-ness, stem cell-like signaling and Warburg metabolism

**Figure 2:**
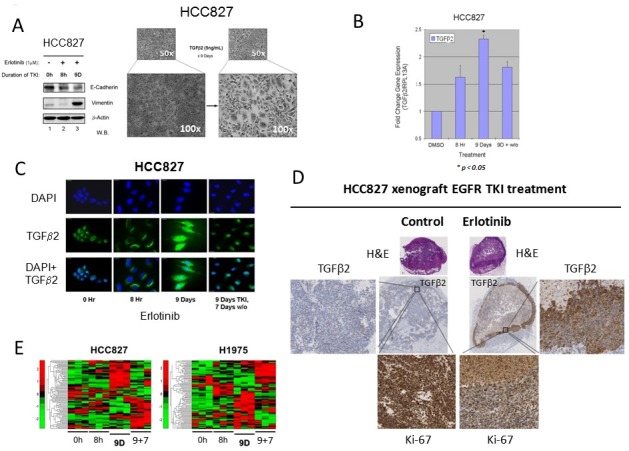
Autocrine TGFβ2 upregulation in lung adenocarcinoma early adaptive drug-escape correlated with EMT and stem cell signaling reprogramming **2A.** HCC827 cells persisting under 9 days of erlotinib treatment displayed progressively downregulated expression of E-cadherin and remarkably increased expression of vimentin at day 9. Bright field microscopic images (*right*) showed HCC827 cells treated with exogenous TGFβ2 (5 ng/mL) for 9 days. **2B.** Q-PCR results verified that the *TGF*β*2* gene expression was upregulated in HCC827 lung adenocarcinoma cells in adaptive escape against erlotinib. HCC827 cells were treated in culture without or with erlotinib for the indicated time durations, and *TGF*β*2* expression was elevated in both 8 hr (1.5 fold) and 9 days (~2.5 fold) cell groups when compared with control cells (DMSO treated) * p < 0.01. **2C.** Immunofluorescence staining of TGFβ2 expression in HCC827 cells treated with erlotinib. Immunocytochemistry shows the TGFβ2 protein expression in cells treated with erlotinib at 3 different time points (8Hr, 9D and 9D+7D washout). DAPI nuclear staining (nuclear stain) is shown in the upper panel, TGFβ2 protein (green) staining alone is shown in the middle panel and both are merged and shown in the bottom panel. Scale bar - 10 μm. **2D.** *In vivo* murine xenograft model of early adaptive drug-escape against erlotinib in HCC827 *EGFR*-mutant lung adenocarcinoma revealed upregulated TGFβ2 and depressed Ki-67 expression. IHC analysis of the post-treatment xenograft tumors using primary antibodies against human TGFβ2 and Ki-67 were performed and shown here (representative images). **2E.** Heat map analysis of stem cell signaling genes expression in *EGFR*-mutant NSCLC.

We next sought to investigate the role of autocrine TGFβ2 in the emergence of the early adaptive drug escape. TGFβ signaling has been well known to be involved in human tumorigenesis although there can be complex patterns of its pathway activation as both tumor promoter and tumor suppressor, often in a tissue context-dependent fashion [16, 17]. TGFβ signaling has also been implicated in inducing EMT and cancer stem cell-like phenotype [18, 19]. Exogenous TGFβ2 treatment (5 ng/ml) in cell culture induced HCC827 cells to adopt an EMT-like phenotype (Figure 2A), correlating with the erlotinib treatment downregulated E-cadherin and induced vimentin expression in cells surviving 9 days of TKI (Figure 2A). Using Q-PCR gene expression assay, we validated that there was an induction of *TGFβ2* mRNA expression (2.5-fold) in the HCC827 cells that were in escape against erlotinib cytotoxicity (up to 9 days), followed by expression reversal after 7 days of drug-washout (Figure 2B). In addition, we also demonstrated that the autocrine TGFβ2 cytokine was adaptively induced at day 9 TKI treatment at the protein level, as seen in the immunofluorescence study, followed by de-escalation after drug-washout (Figure 2C). It is intriguing to note that there was a TGFβ2 nuclear translocation from cytoplasmic compartment in the early adaptive TKI escaping cells evident at 9 days erlotinib inhibition, which was partially reversed after 7 days of drug-washout (Figure 2C). As reported recently, the early adaptive drug-escaping HCC827 cells evading erlotinib inhibition can be observed in an *in vivo* xenograft TKI treatment model [13]. We identified that there was upregulation of BCL-2/BCL-xL prosurvival signaling and p-STAT3 activation in HCC827 cells in adaptive escape against erlotinib, primarily localizing along the peripheral rind of the tumor xenograft as in the TKI evading cells [13]. We now identified that the HCC827 xenograft under *in vivo* erlotinib inhibition for 4 days resulted in an induction of autocrine TGFβ2 expression intratumorally in the adaptive TKI-evading cells (Figure 2D), associating with a corresponding suppression of the proliferative marker Ki-67 expression (Figure 2D). Our results support the notion that the early adaptive drug escape involves a predominantly cellular quiescence state. Moreover, our gene expression clustering analysis identified that in both HCC827 cells and H1975 cells treated under the corresponding EGFR TKI, there was an adoptive stem cell signaling gene expression reprogramming evident during the early drug escape cell state (day 9 under TKI) (Figure 2E).

**Figure 3:**
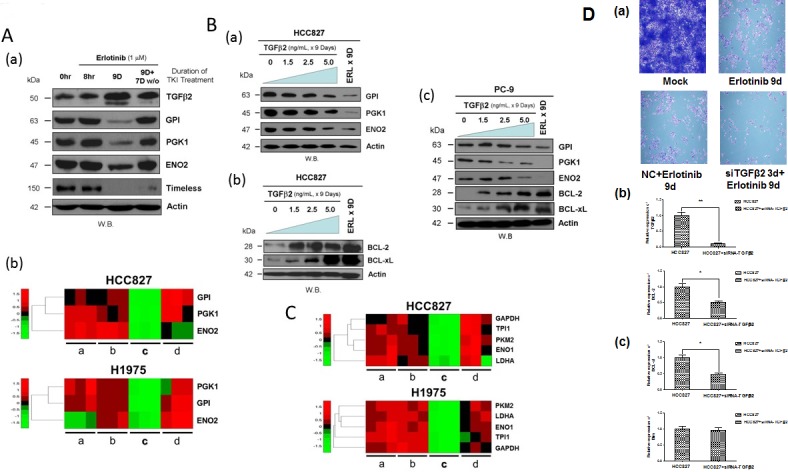
Downregulation of glycolytic regulatory enzymes expression through TGFβ2 signaling to promote pro-survival mitochondrial-priming **3A.** Induction of autocrine TGFβ2 cytokine expression in HCC827 cells persisted after 9 days of erlotinib treatment undergoing adaptive drug-escape, correlated with downregulated expression of the key glucose metabolism regulatory enzymes *GPI*, *PGK1* and *ENO2*, as well as the cell cycle progression regulator *TIMELESS*. *(a):* HCC827 cells were treated without (0 hr) or with erlotinib for 8 hr, 9 days, and 9 days of TKI followed by 7 days of drug washout. Actin (bottom panel) was included as loading control. *(b):* Gene expression heat map signature showing adaptive suppression in gene expression of glucose metabolism regulatory Warburg genes *GPI*, *PGK1* and *ENO2* in HCC827 cells (*upper panel*) treated with erlotinib and H1975 cells (*lower panel*) treated with CL-387,785. TKI treatment conditions: *a,* 0 hr (untreated control); *b,* 8 hr; *c*, 9 days and *d*, 9 days of TKI followed by 7 days of drug washout. **3B.** Immunoblot analysis of the effect of TGFβ2 on HCC827 and PC-9 cells in the expression levels of the glucose metabolism regulatory enzymes: GPI, PGK1, ENO2, and the mitochondrial prosurvival marker BCL-2/BCL-xL. *(a)* HCC827 cells were treated with exogenous TGFβ2 (5 ng/ml) for 9 days, at increasing concentration of the cytokine as indicated for immunoblotting. *(b & c)* HCC827 and PC-9 cells under similar treatment conditions as above were subjected to immunoblotting. **3C.** Gene expression heat map signature of suppressed Warburg glycolytic enzyme genes in early adaptive *EGFR*-mutant NSCLC cells under targeted EGFR-TKI treatment. HCC827 cells treated with erlotinib (*upper panel*) and H1975 cells treated with CL-387,785 (*lower panel*). *PKM2*, pyruvate kinase M2; *LDHA*, lactate dehydrogenase A; *ENO1*, enolase 1; *TPI1*, triosephosphate isomerase 1; *GAPDH*, glyceraldehyde-3-phosphate dehydrogenase. TKI treatment conditions: *a,* 0 hr (untreated control); *b,* 8 hr; *c*, 9 days and *d*, 9 days of TKI followed by 7 days of drug washout. **3D.** (*a*) SiRNA-specific silencing of TGFβ2 (3 days) in erlotinib-treated HCC827 cells for 9 days compared with the untreated, 9-day treated erlotinib and the mock control-treated HCC827 cells. (*b & c*) siRNA knockdown of TGFβ2 shows a significant reduction in mRNA expression of TGFb2, BCL-2, and BCL-xL. BIM showed no significant difference in expression upon siRNA silencing of TGFβ2.

Immunoblotting analysis further verified at the protein levels the modulating effects of erlotinib inhibition on expression of various markers of the Warburg effect (glucose metabolism genes) and cell cycle gene, identified from the gene expression microarray analysis in HCC827 cells (Figure 3A). Here, we found that the TKI-modulated expression of the glycolytic metabolic enzymes GPI, PGK1 and ENO2, and the cell cycle progression regulator TIMELESS paralleled the expression pattern as observed in the transcriptomic analysis. All these markers were found consistently downregulated in the early adaptive drug-evading cells, but readily reversible upon TKI-washout (Figure 3A-a, 3A-b). Next, we asked if direct TGFβ2 effects on HCC827 cells would recapitulate that observed under erlotinib inhibition as above. We found that exogenous TGFβ2 was sufficient to inhibit, in a concentration dependent fashion, the expression levels of the key glycolytic metabolic enzymes involved in the “Warburg effect”, GPI, PGK1 and ENO2 (Figure 3B-a). Importantly, we also validated that exogenous TGFβ2 is sufficient to induce the intrinsic mitochondrial prosurvival priming with enhanced expression of the markers BCL-2 and BCL-xL in the drug-evading HCC827 cells (Figure 3B-b). Similar results were obtained and validated in another *EGFR*-mutant lung adenocarcinoma cell line PC-9 (Figure 3B-c). Finally, we extended our gene expression signature analysis into other well-recognized Warburg metabolism regulatory genes *PKM2*, *LDHA*, *ENO1*, *TPI1* and *GAPDH*, and found that they were all adaptively downregulated in expression within the *EGFR*-mutant NSCLC cells under EGFR-TKI drug escape (Figure 3C). Finally, to directly assess the functional significance of TGFβ2 in the emergence of early adaptive resistant cells, we performed RNAi-silencing of TGFβ2 in HCC827 cells subjected to precision treatment with erlotinib for 9 days (Figure 3D-a). Upon TGFβ2 RNAi knockdown, there was a significant impact leading to downregulation of both BCL-2 and BCL-xL expression, while there was no difference in the expression of BIM (Figure 3D-b & 3D-c).

### Remarkable global metabolome reprogramming of key bioenergetics pathways in HCC827 lung adenocarcinoma cells during early adaptive drug escape

We further adopted a mass-spectrometry based global metabolomics platform to quantitatively profile the expression of metabolites in HCC827 cells under erlotinib treatment. Here, we demonstrated that there was a dramatic metabolome-wide adaptive reprogramming of key bioenergetics pathways in the HCC827 cells undergoing drug escape against erlotinib. The present dataset comprises a total 356 compounds of known identity (named biochemicals). Following log transformation, normalization to Bradford protein concentration and imputation with minimum observed values for each compound, Welch's two-sample *t*-test was used to identify biochemicals that differed significantly between experimental groups. A summary of the numbers of biochemicals that achieved statistical significance (*p*≤0.05), as well as those approaching significance (0.05 < *p* < 0.10), is shown in tables S3.

#### Glucose metabolism and glutaminolysis reprogramming (Figure 4A)

**Figure 4A:**
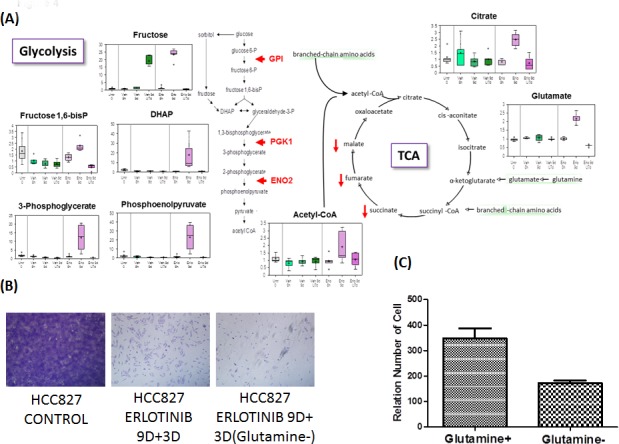
Glucose metabolism, TCA cycle and glutaminolysis reprogramming. **4B.** Erlotinib-treated HCC827 cells were treated with and without glutamine for 9 days followed by three days of no treatment. **4C.** Relative number of viable early adaptive resistant cells without glutamine was significantly reduced compared with the cells treated with glutamine.

Significant alterations were observed in glycolytic and TCA cycle intermediates and glutaminolysis within the early onset drug resistant tumor cells. In cells treated with erlotinib, the glucose flux through the glycolytic and tricarboxylic acid (TCA) pathways and glutaminolysis was apparently reduced after the TKI treatment for 9 days and 9 days TKI + 7 days drug-washout. Treatment with erlotinib in 8 hr did not significantly affect the glycolytic intermediates whereas an increase was observed in levels of glutamine, glutamate and the TCA cycle intermediates such as citrate, succinate and its carnitine derivate succinyl carnitine, fumarate and malate as compared to vehicle-treated controls. This increase is suggestive of increased TCA cycle flux and is consistent with the observed increase in glucose utilization. A large effect in 9 days of erlotinib treatment was observed as there were significant elevations in glucose, fructose, several glycolytic intermediates, and lactate. Furthermore, the survivor cells in drug-escape exhibited significantly reduced levels of succinate, fumarate and glutamate, despite significant increases in glutamine and glutamate, suggestive of reduced TCA cycle activity and decreased glutaminolysis. These changes are consistent with a large reduction in glucose utilization after 9 days of erlotinib inhibition, supporting the notion that the early adaptive drug escape cells adopt a cellular-metabolic quiescence-like state [13]. After an additional 7 days drug-washout period following 9 days of erlotinib treatment, glucose utilization was resumed and may be slightly greater than that observed in vehicle-treated control while the TCA cycle activity was also reversed and became similar to that observed in untreated control cells. We next asked if the adaptive drug-resistant tumor cells could be newly addicted to glutamine. To test this, HCC827 cells were cultured in the presence or absence of glutamine, under EGFR TKI treatment with erlotinib for 9 days followed by 3 washout days. Glutamine-withdrawal led to significantly less viable early adaptive erlotinib-resistant cells suggesting that the cells adopted a degree of glutamine-addiction during the drug-induced metabolic reprogramming (Figure 4B and 4C).

#### Branched-chain amino acid catabolism reprogramming (Figure 5, left; and Figure S2)

Degradation products formed from the catabolism of the branched-chain amino acids (BCAA) valine, isoleucine and leucine are used for anaplerotic contribution to the TCA cycle. Despite minimal changes in levels of TCA cycle intermediates; there was a reduction in BCAA carnitine conjugates after 8 hours of erlotinib treatment. However, 9 days of erlotinib significantly increased valine, isoleucine, and leucine and their carnitine derivatives, suggestive of reduced entry into the TCA cycle which is consistent with reduced glucose utilization and TCA cycle intermediates in cells evading erlotinib therapy. After the 7 days erlotinib withdrawal period, levels of BCAAs were significantly reduced and the carnitine conjugates remain slightly elevated, suggestive of reduced oxidation of these amino acids for energy and potential increased utilization for protein synthesis.

**Figure 5:**
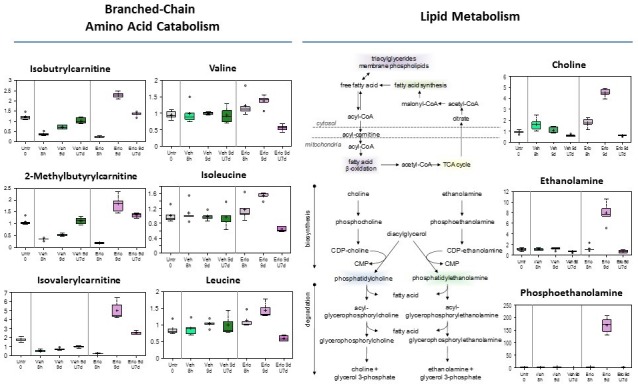
Branched-chain amino acid catabolism, and Lipid metabolism reprogramming in early adaptive drug-resistant HCC827 EGFR-mutant cells under erlotinib inhibition

#### Lipid metabolism reprogramming (Figure 5, right, table S4)

One of the most striking and consistent changes observed was a significant increase in many essential, medium-chain and long-chain fatty acids found in cells that persisted after 9 days of erlotinib treatment. Significant elevations in the fatty acid synthesis precursors - citrate, acetyl CoA and its carnitine derivative acetylcarnitine, are suggestive of a blockage in fatty acid synthesis; therefore, increased fatty acid synthesis reflects increased uptake and/or decreased synthesis of membrane phospholipids as several precursors in the biosynthetic pathway such as choline, ethanolamine and phosphoethanolamine were significantly elevated. Furthermore, cell membranes are highly enriched in sphingolipids and the significant reduction in palmitoyl sphingomyelin and stearoyl sphingoymyelin are supportive of the notion of reduced membrane synthesis with 9 day treatment of erlotinib. This is also consistent with a proliferative quiescence-state observed at 9 day treatment of erlotinib [13]. Conversely, the opposite findings of reduced levels of many free fatty acids and decreased precursors of membrane lipid synthesis with 9 days erlotinib + 7 days drug-washout are consistent with the notion of an adaptive increased proliferation and growth as a result of the removal of therapeutic stress.

#### Inflammation and endocannabinoids reprogramming (Figure S3)

A large increase in arachidonate (20:4n6) and two associated eicosanoids, 13,14-dihydro-15-keto-prostaglandin E2 and 13,14-dihydro-15-keto-prostaglandin E2, were observed exclusively with 9 days of erlotinib treatment in HCC827 cells. Interestingly, short-term increases in oleic ethanolamide, palmitoyl ethanolamide, N-stearoyl taurine, N-palmitoyl taurine, and oleoyltaurine were observed with 8 h of erlotinib treatment, followed by significant reductions in all endocannabinoids except palmitoyl ethanolamide with 9 day of TKI therapy. In addition to effects on tumor cell proliferation and apoptosis, N-acyl taurines are known to activate the transient receptor potential (TRP) family of calcium channels. One member of this family, TRPV4, is involved in regulation of osmotic pressure, and several major osmolytes including glycerophosphorylcholine (GPC), myo-inositol, and taurine were significantly elevated in cells evading erlotinib treatment after 9 days. These findings are reflective of a cell state in an attempt at protection against hypertonicity and osmotic stress.

#### Neuropeptide signature (Figure S4)

Synthesis of N-acetylaspartate (NAA) and N-acetyl-aspartyl-glutamate (NAAG) was found selectively increased only after 9 days of treatment with erlotinib. Serotonin (5HT) production was significantly greater in cells treated with erlotinib for 8h and 9 days compared to vehicle-treated controls, but after the 7 days washout period, serotonin levels were similar in both treatment groups.

#### Methionine metabolism and oxidative stress (Figure S5)

Depletion of the tripeptide glutathione and associated increases in oxidative stress are a hallmark of many cancers. Indeed, in HCC827 cells treated with vehicle and erlotinib, there was a reduction in both oxidized glutathione (GSSG) and reduced glutathione (GSH) over time as compared to untreated control. Interestingly, there was a large increase in levels of cysteine, the rate-limiting biochemical for synthesis of glutathione, after 9 days and 9 days plus 7 days washout of vehicle treatment, suggestive of a “blockage” in glutathione synthesis. Nine days of erlotinib treatment induced a massive depletion of cysteine with concurrent increases in methionine, taurine, and ophthalmate, suggestive of a shunting of cysteine toward alternative pathways other than glutathione synthesis. As mentioned previously, increased taurine levels may be related to endocannabinoid synthesis and/or osmoregulation, and increased ophthalmate, a glutathione-like biochemical synthesized by the same enzymes as glutathione, indicates that the reduction in cysteine was the limiting factor for glutathione synthesis after 9 days of erlotinib therapy. Similarly, depletion of cysteine induced by 9 days of erlotinib therapy was likely responsible for the significant reduction in the oxidative stress marker cysteine-glutathione disulfide, as opposed to an actual improvement in oxidative environment.

#### Arginine metabolism and polyamines (Figure S6)

Treatment with erlotinib significantly affected arginine metabolism after 9 days of therapy, as conversion of arginine to citrulline by inducible nitric oxide synthase (iNOS) may be reduced due to a significant elevation in dimethylarginine (ADMA/SDMA), a potent endogenous inhibitor of iNOS activity. Increased dimethylarginine may be indicative of elevated oxidative stress, as levels of carnosine, a dipeptide derivative that functions as an antioxidant, were selectively elevated at this time point as well. In addition, alternative pathways for metabolism of arginine were affected by 9 days of erlotinib treatment as creatine and creatinine were elevated, ECM remodeling was altered as evidenced by increased proline and pro-hydroxy-pro, and production of the polyamines spermidine and spermine was significantly increased.

### TGFβ2-BCL-2/BCL-xL linked mitochondrial metabolically-regulated prosurvival cascade in early adaptive drug escape can be therapeutically targeted

**Figure 6:**
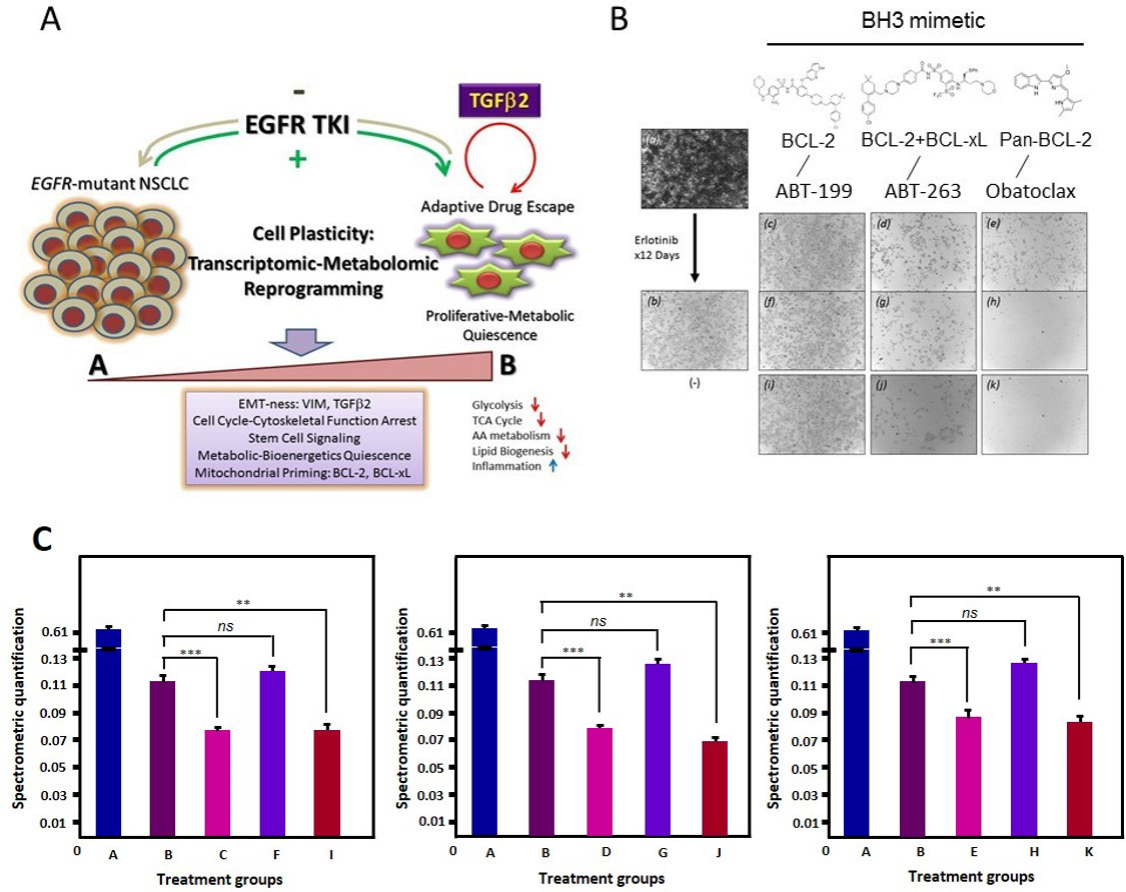
Early adaptive transcriptomic-metabolomic cellular reprogramming under EGFR-TKI precision therapy to promote resistant drug-escape **6A,** The schematic diagram highlights the central role of autocrine TGFβ2 in *EGFR* oncogene-addicted NSCLC. **6B,** Cells were then treated with erlotinib (1 μM) for up to 9 days, with intermittent inhibitor replenishment every 3 days. After that, cells were further treated for 3 additional days using (*a*) diluent alone, (*b*) erlotinib alone, ABT-199 without (*c*) or with (*f*) continuing erlotinib, ABT-263 without (*d*) or with (*g*) continuing erlotinib, obatoclax without (*e*) or with (*h*) continuing erlotinib. HCC827 cells were also treated concurrent at the beginning of the experiment using erlotinib (1 μM) plus the respective BH3 mimetics (2 μM) as above (*i*: ABT-199, *j*: ABT-263, and *k*: obatoclax). **6C,** Spectrometric quantification of the different groups including the untreated (*A*), treated with erlotinib (*B*: 12 days 1 μM Erlotinib), treated with erlotinib and ABT-263 (*C:* 12 days 1 μM Erlotinib + 3 days 2 μM ABT-263; *D:* 9 days 1 μM Erlotinib + 3 days 2 μM ABT-263; *E:* 12 days 1 μM Erlotinib + 12 days 2 μM ABT263), treated with erlotinib and ABT-199 (*F:* 12 days 1 μM Erlotinib + 3 days 2 μM ABT-199; *G:* 9 days 1 μM Erlotinib + 3 days 2 μM ABT-199; *H:* 12 days 1 μM Erlotinib + 12 days 2 μM ABT-199) and treated with Obatoclax (*I:* 12 days 1 μM Erlotinib + 3 days 2 μM Obatoclax; *J:* 9 days 1 μM Erlotinib + 3 days 2 μM Obatoclax; *K:* 12 days 1 μM Erlotinib + 12 days 2 μM Obatoclax). (ns - not significant; ** *p* < 0.002; *** *p* < 0.001).

Using BH3 mimetics [20] with differential mitochondrial inhibitory activities, i.e. ABT-263 [21], ABT-199 [22, 23], and obatoclax [24, 25], we further tested the hypothesis that the EGFR TKI induced, TGFβ2-mediated mitochondrial prosurvival priming can be therapeutically targeted to prevent or eradicate the early adaptive drug escape in *EGFR*-mutant NSCLC. In an *in vitro* cell survival assay in HCC827 cells under treatment with (i) erlotinib alone, and (ii) erlotinib plus BH3 mimetic - either ABT-263 (dual BCL-2/BCL-xL potent), ABT-199 (BCL-2 > > BCL-xL potent), or obatoclax (pan-BCL-2 family potent) inhibitor (Figure 6). The BH3 mimetic was added either at day 0 concurrently with erlotinib or at day 9 after erlotinib induced mitochondrial priming emergence, with or without ongoing concurrent erlotinib for 3 additional days. Our results show that it is more efficacious to adopt a concurrent approach to combine EGFR TKI erlotinib with a dual BCL-2/BCL-xL BH3 mimetic with ABT-263 from the start of therapy to preemptively prevent the emergence of early adaptive drug-escape cells against erlotinib (Figure 6B: g, h, j, k). Of note, ABT-199 with highly specific potent activities against BCL-2 only but not BCL-xL, did not show significant efficacy here (Figure 6B: c, f, i & Figure 6C), further validating our previous observation in RNAi knockdown studies that BCL-xL is necessary therapeutic partner with BCL-2 to effectively inhibit the early adaptive erlotinib resistant HCC827 cells [13]. Interestingly, the pan-BCL-2 family inhibitor obatoclax yielded a superior efficacy in both preventing the emergence of early adaptive drug escape as well as eradication of the mitochondrially primed drug-persisting cells emerged after 9 days of prior erlotinib inhibition.

## DISCUSSION

The challenge of clinical tumor resistance remains a major bottleneck to meaningfully impact long term survival outcome of precision therapy in *EGFR*-mutant lung cancer. We and others have recently identified a novel paradigm of early emergence of adaptive subpopulation of *EGFR*-mutant lung cancer cells in escape against the corresponding targeted inhibitor from the parental drug-sensitive cell population within a very “early” time-window only days after therapy initiation [13, 26]. These early adaptive drug evading survivors likely serve as the founder population as minimal residual disease under therapeutic pressure, which ultimately evolve into clinically detectable resistant progressive disease on therapy late in the future [13, 26, 27].

The early adaptive drug-evading HCC827 cells also showed downregulated E-cadherin and upregulated expression of EMT inducer protein marker vimentin, besides TGFβ2. The cell state molecular switch from epithelial to mesenchymal phenotype has been associated with activation of signaling and transcription factors primarily involving TGFβ signaling. EMT induction has been demonstrated to play key role in late clinical acquired EGFR inhibitor resistance [10] and as supported by our study results, may indeed have initiated very early in drug escape process within the continuum of clinical drug resistance evolution. Altered cellular metabolism is now known to often exist in human cancers [28, 29], playing a direct tumorigenic role in the process. Otto Warburg first observed the phenomenon in the 1920s, now known as the “Warburg effect” or “aerobic glycolysis”. Normal cells depend predominantly on mitochondrial oxidative phosphorylation to generate ATP and energy from glucose. In contrast, cancer cells can rewire their metabolic programs in preference to metabolize glucose in a larger part by glycolysis, resulting in increased glucose consumption and lactate production even in the presence of ample oxygen [14, 30, 31]. Of particular interest, our current study results notably identified a novel adaptive metabolomic reprogramming under molecularly targeted therapeutics stress resulting in globally suppressed cellular metabolism including glycolytic pathway, TCA cycle pathway, branched chain amino acids metabolism, lipid biogenesis, and enhanced inflammatory metabolism, evidently in an overall effort to promote tumor cells survival and therapeutic escape. The practical implication is that these adaptive *EGFR-*mutant lung cancer cells that survive erlotinib in escape would also evade detection by clinical ^18^FDG-PET scanning due to their suppressed glucose metabolism, forming therefore the basis of radiographic complete response (CR) or near-CR despite the presence of minimal residual tumor disease in therapeutic escape. These quiescent minimal residual molecular drug-resistant tumor cells in drug-escape could ultimately evolve with time into full proliferative resistant progressive disease late in the future.

Our study findings not only established the relevance of autocrine TGFβ2 in early adaptive EGFR-TKI drug-escape in *EGFR*-mutant NSCLC, but also highlight for the first time, to our best knowledge, that TGFβ2 signaling links with the mitochondrial BCL-2/BCL-xL prosurvival cascade in the context of a profound cellular metabolome reprogramming. We recently presented both *in vitro* and *in vivo* evidence [13] to support the notion that the druggable BCL-2-family signaling in the intrinsic mitochondrial programmed-cell death pathway may represent a central mechanism in EGFR-TKI-induced mitochondrial priming [32, 33] to achieve new prosurvival dependence in promoting and sustaining early adaptive drug escape against the TKI [13, 34]. We also presented the first *in vivo* proof-of-concept evidence to support the efficacy of combination BCL-2/BCL-xL BH3 mimetic with EGFR TKI in circumventing early adaptive drug resistance and thus resulting in durable tumor response in EGFR-mutant NSCLC precision therapy [13]. Here, we show that through shRNA knockdown studies, TGFβ2 directly mediates the survival of the adaptive EGFR-TKI drug-escaping tumor cells, and that it also directly regulates the mitochondrial BCL-2/BCL-xL expression downstream, but not BIM. Moreover, our omics profiling study revealed profound genome- and metabolome-wide cellular reprogramming within *EGFR*-mutant NSCLC cells undergoing early drug-escape, mediated by TGFβ2 autocrine signaling. Importantly, we further demonstrated that such large scale omics landscape alteration represents a highly reversible adaptation to the therapeutic stress, hence highlighting the critical importance of gene expression changes and metabolic regulation in precision therapy tumor resistance, a perspective that was not well appreciated in the past. Our current study also provided evidence to suggest that BCL-xL is a key therapeutic target, rather than merely BCL-2 alone, in the process of early adaptive EGFR-TKI drug escape emergence. Hence, it is crucial to adopt at least dual BCL-2/BCL-xL BH3-mimetic (ABT-263), or even pan-BCL-2 family BH3-mimetic (obatoclax) inhibitor [20, 24, 25, 35–37], in order to effectively prevent or eradicate early adaptive mitochondrially-primed tumor escape. Moreover, the early adaptive drug-resistant cells were also found to be vulnerable to glutamine deprivation, suggesting that they were relatively glutamine-addictive. Glutamine is known to serve as a crucial nitrogen donor for nucleotide synthesis. MYC has also been shown to play as a key promotional factor to the metabolic process of converting glutamine into glutamic acid, which ultimately would be converted to lactic acid towards the end of the metabolism. According to the Warburg effect, the cancer cell undergo oncogenic activation of glucose uptake and metabolism, often induced by constitutively activated PI3K signal path. This Warburg's metabolism leads to resultant cellular secretion of excess glycolytic metabolites in the form of lactic acid. This apparent inefficient metabolism of glucose is observed in parallel in some human tumors implicated to display “glutamine-addiction”, and that the cellular survival is dependent on the presence of exogenous glutamine [38]. Previous reports suggest cell death induced by glutamine-depletion is mediated through the intrinsic mitochondrial apoptotic pathway, as it could be rescued by BCL-2/BCL-xL overexpression, or a dominant-negative caspase-9 [39].

To this end, it is also of interest to identify the autocrine TGFβ2 signaling as the key cellular reprogramming mediator within the early adaptive drug escaping cells. TGFβ signaling has been shown to play important roles in human tumorigenesis with relatively complex tissue-specific and stromal context-specific signaling effects [40]. Its activation can be highly contextual in nature, both pivotal in early tumorigenesis process as “tumor suppressor” and in late stages of tumorigenesis and progression as “tumor promoter” [41]. Furthermore, the role of TGFβ signaling has been much better understood as a stromal influence in cancer development [42]. TGFβ signaling has been implicated as a link to increased ERK signaling and TKI resistance in PC-9 NSCLC cells through loss of *MED12* [43–45]. Here, our studies suggest that specifically TGFβ2 signaling activation is autocrine in nature, and mimics a “tumor suppressor” effect in short-term perspective to suppress tumor cell metabolism, proliferation, and motility in the early cellular adaptive drug escape against erlotinib, resulting in a long term overall tumor survival promotion that would ascertain the ultimate persistence, proliferative resistance and survival of the NSCLC cells throughout the therapeutic stress.

In conclusion, our study revealed a profound transcriptomic-metabolomic cellular reprogramming during the early-onset drug escape of *EGFR*-mutant NSCLC cells against precision therapy, with resultant significant mitochondrial prosurvival priming. This was correlated with an autocrine TGFβ2 upregulation, increased EMT-ness and cancer stemness, arrested cell cycle and cytoskeletal function, and remarkable adaptive transcriptome-metabolome-bioenergetics alterations (Figure 6). It would be attractive to further validate these findings in prospective serial rebiopsy study in *EGFR*-mutant patients under EGFR-TKI therapy, including the early time-window in the midst of tumor response (1^st^ week to 1-2 months post-therapy), using comprehensive transcriptomic profiling analysis [43]. The key metabolic deregulation involved global reduction in glucose metabolism, TCA cycle activity, glutaminolysis, oxidation of branched-chain amino acids, and reduced synthesis of fatty acids and membrane lipid components. This overall reduction in energy metabolism was accompanied by significant increases in several cofactors, including ADP, ATP, NAD+, NADH, NADP+, and coenzyme A, likely due to reduced energy demands and utilization. Finally, we show that the early adaptive drug-resistant EGFR-mutant tumor cells under reprogrammed transcriptomic/metabolomics profiles can be secondarily targetable in principle, and are vulnerable to TGFβ2 inhibition, and glutamine-depletion. Last, we also provide further evidence to the efficacy of combined EGFR-TKI and BCL-xL-targeting BH3 mimetic [46] co-inhibitory strategy to dampen the emergence of early adaptive drug escape in *EGFR*-mutant NSCLC precision therapy. Our results further nominated the prosurvival mitochondrial marker BCL-xL as an important and necessary co-target in addition to BCL-2 itself, to achieve optimal therapeutic efficacy to eradicate the emergence of adaptive EGFR-TKI evasion. Hence, it would now be feasible to derive rationally designed co-inhibitory strategy to prevent or overcome early adaptive drug-resistance emergence targeting the TGFβ2-mediated mitochondrial bioenergetics reprogrammed prosurvival attractive paths at the nodal points of BCL-xL and glutamine metabolism. Further validation studies to test these secondary combination strategies to combat EGFR-TKI drug resistance emergence promise to further optimize cancer precision therapy by enhancing the duration of therapy response and impacting long term clinical outcome.

## MATERIALS AND METHODS

### Chemicals, cell culture, immunoblotting and immunofluorescence

EGFR inhibitor (reversible) erlotinib was prepared as previously described [47, 48]. EGFR inhibitor (irreversible) CL-387,785 was obtained from EMD-Calbiochem (Cambridge, MA). The BH3 mimetics, ABT-263 (navitoclax), ABT-199 and obatoclax were purchased from Selleck, Inc. (Houston, TX). ABT-263 is a potent inhibitor of BCL-xL, BCL-2 and BCL-w with K_i_ of ≤ 0.5 nM, ≤1 nM and ≤1 nM, but binds more weakly to MCL-1 and A1. ABT-199 (GDC-0199) is a potent BCL-2-selective inhibitor with K_i_ of < 0.01 nM, > 4800-fold more selective *versus* BCL-xL and BCL-w, and no activity to MCL-1. Obatoclax (GX15-070) is a pan-BCL-2 family antagonist with K_i_ of 0.22 μM.

Lung cancer cell lines were obtained directly from American Type Culture Collection (ATCC) and grown under standard cell culture conditions. Cell lines characterization and authentication were performed by the ATCC Molecular Authentication Center, using COI for interspecies identification and STR analysis (DNA fingerprinting) for intraspecies identification. SDS-PAGE and Western blotting were performed as previously described [48, 49]. The primary antibodies used are as follows: BCL-2, BCL-xL, (both from Zymed), TGFβ2 (ab66045, Abcam, Cambridge, MA), GPI (H-300, SC-33777, Santa Cruz), PGK1 (PA5-13973, Thermo Fischer Scientific, Inc., Waltham, MA), ENO2 (LS-CC88776, LifeSpan BioSciences, Albuquerque, NM), TIMELESS (3709-1, Epitomics, Burlingame, CA) and actin (I-19, Santa Cruz).

For cell survival assays in the studies of cells under 9 days of pretreatment under targeted inhibitors, the indicated inhibitors in the culture media were replenished at least every 2-3 days prior to cell harvesting at the end of the inhibitory culture for subsequent cellular assays. Crystal violet cell survival staining assay was performed as previously described [13]. For immunofluorescence staining, HCC827 cells of different groups of 0hr, 8hr, D9 and D9+7 treated with erlotinib in Nunc chamber slides were fixed in 4% formaldehyde followed by permeabilization with 0.2% Tr-X 100 for an hour at 4°C. The cells were then washed with PBS three times and blocked for an hour in 5% normal donkey serum for 1 hr at room temperature. The cells were then incubated with TGFβ2 primary antibody overnight at 4°C, washed with PBS three times and then incubated with AF488 anti-rabbit secondary antibody for an hour at room temperature. Cells were then washed three times with PBS. The slides were then observed and recorded under fluorescence microscopy.

### Transcriptomic profiling of gene expression: affymetrix microarray analysis

Total RNA samples were extracted from triplicate cell culture samples from 0hr (Untreated control), 8hr, Day 9 and Day 9+7 (9 days of TKI followed by 7 days of drug wash-out) for the two NSCLC cell lines namely, HCC827 and H1975, treated with erlotinib and CL-387,785 respectively, according to standard procedures. The samples were then submitted to the Gene Expression and Genotyping Facility of the Case Comprehensive Cancer Center for transcriptomic profiling. Sample aliquots (150 ng) were prepared for microarray using Ambion's WT expression kit (Cat# 4411974) according to the manufacturer's instructions. Total RNA samples were extracted from triplicate cell culture samples from 0hr (Untreated control), 8hr, Day 9 and Day 9+7 (9 days of TKI followed by 7 days of drug wash-out) for the two NSCLC cell lines namely, HCC827 and H1975, treated with erlotinib and CL-387,785 respectively, according to standard procedures. The samples were then submitted to the Gene Expression and Genotyping Facility of the Case Comprehensive Cancer Center for transcriptomic profiling and analysis (details see Supplemental Methods).

### Quantitative real-time-polymerase chain reaction (Q-RT-PCR)

Gene expression of TGFβ2 was verified by quantitative real-time polymerase chain reaction (Q-RT-PCR). Total RNA extractions were performed with an RNeasy Plus Mini kit (Qiagen). cDNA were synthesized from RNA samples with an iScript cDNA synthesis kit (BIO-RAD). After cDNA synthesis, PCR reactions were setup with an iQ SYBR Green Supermix kit (BIO-RAD). Relative quantification was done on Fast Real-Time PCR system (ABI 7500). Using the comparative threshold cycles (CT) method, the quantification normalized to untreated cells were performed. Experiments were performed in triplicates.

### Exogenous TGFβ2 treatment of HCC827 cells

HCC827 and PC-9 cells were treated with exogenous TGFβ2 in different concentrations of 1.5 ng/mL, 2.5 ng/mL and 5 ng/mL for 9 days. Cell lysates were prepared from the different concentrations of TGFβ2 treatment and immunoblotting was performed using these lysates probed with different anti-apoptotic and metabolic primary antibodies. The cells were also observed under the microscope at the end of the treatment.

### Preparation and transfection of siRNAs targeting TGFβ2

In this study, siRNAs targeting *TGFβ2* (Ruibo, Guangzhou, China) were employed in HCC827 cells for knockdown experiments. The sequences of each siRNA pair were as follows: CGGAGGTGATTTCCATCTA, CGACAGCAAAGTTGTGAAA, CTATAAAGTCCACTAGGAA. siRNAs were transfected into 90-100% confluent HCC827 cells at the final concentration of 10 nM using standard methods. Two to three days after transfection, the efficacy of knockdown was assessed by QPCR.

### Glutamine deprivation of HCC827 cells

HCC827 were treated with and without glutamine, which were treated with erlotinib for 9 days followed by 3 days of drug-washout. The cells were then fixed and assayed under the microscope at the end of the treatment, with crystal violet cell survival staining. The stained viable cells were quantified and plotted for the comparison between glutamine-treated and glutamine-withdrawn groups.

### Metabolomics profiling

Global biochemical profiling was performed using HCC827 cell samples, the groups being untreated cells at time 0 and cells treated with erlotinib (1 μM) or DMSO (dimethyl sulfoxide) vehicle at 3 time points of 8 hr, Day 9 and Day 9+7 (9 days erlotinib/DMSO, followed by 7 days washout), with 5 replicate in each treatment group. All 35 samples were extracted using the automated MicroLab Star^®^ system from Hamilton Company (Metabolon Inc., Durham, NC). Recovery standards were added before the extraction process for QC purposes. Sample preparation was performed using a proprietary series of organic and aqueous extractions that allow maximum recovery of small molecules. Details of the methods of metabolomics profiling and analysis is described in the Supplemental Methods and Materials.

### *In vivo* xenograft model of HCC827 NSCLC

HCC827 and their corresponding murine xenograft models without or with erlotinib treatment for 4 days (n = 6/group), were established as previously described, according to institution approved protocols and guidelines [13, 49]. The studies were conducted in accordance with the NIH Guide for the Care and Use of Laboratory Animals. Hematoxylin & eosin (H&E) staining and immunohistochemical (IHC) analysis of the tumor xenograft was performed in the Image Core, Cleveland Clinic, using anti-human TGFβ2, and anti-human Ki-67 primary antibodies using standard methods.

## SUPPLEMENTARY MATERIAL











## References

[1] Keedy VL, Temin S, Somerfield MR, Beasley MB, Johnson DH, McShane LM, Milton DT, Strawn JR, Wakelee HA, Giaccone G (2011). American Society of Clinical Oncology provisional clinical opinion: epidermal growth factor receptor (EGFR) Mutation testing for patients with advanced non-small-cell lung cancer considering first-line EGFR tyrosine kinase inhibitor therapy. J Clin Oncol.

[2] Mok TS, Wu YL, Thongprasert S, Yang CH, Chu DT, Saijo N, Sunpaweravong P, Han B, Margono B, Ichinose Y, Nishiwaki Y, Ohe Y, Yang JJ (2009). Gefitinib or carboplatin-paclitaxel in pulmonary adenocarcinoma. N Engl J Med.

[3] Zhou C, Wu YL, Chen G, Feng J, Liu XQ, Wang C, Zhang S, Wang J, Zhou S, Ren S, Lu S, Zhang L, Hu C (2011). Erlotinib *versus* chemotherapy as first-line treatment for patients with advanced EGFR mutation-positive non-small-cell lung cancer (OPTIMAL, CTONG-0802): a multicentre, open-label, randomised, phase 3 study. Lancet Oncol.

[4] Tsao MS, Sakurada A, Cutz JC, Zhu CQ, Kamel-Reid S, Squire J, Lorimer I, Zhang T, Liu N, Daneshmand M, Marrano P, da Cunha Santos G, Lagarde A (2005). Erlotinib in lung cancer - molecular and clinical predictors of outcome. N Engl J Med.

[5] Wang W, Li Q, Takeuchi S, Yamada T, Koizumi H, Nakamura T, Matsumoto K, Mukaida N, Nishioka Y, Sone S, Nakagawa T, Uenaka T, Yano S (2012). Met kinase inhibitor E7050 reverses three different mechanisms of hepatocyte growth factor-induced tyrosine kinase inhibitor resistance in EGFR mutant lung cancer. Clin Cancer Res.

[6] Oxnard GR, Arcila ME, Sima CS, Riely GJ, Chmielecki J, Kris MG, Pao W, Ladanyi M, Miller VA (2011). Acquired resistance to EGFR tyrosine kinase inhibitors in EGFR-mutant lung cancer: distinct natural history of patients with tumors harboring the T790M mutation. Clin Cancer Res.

[7] Sequist LV, Waltman BA, Dias-Santagata D, Digumarthy S, Turke AB, Fidias P, Bergethon K, Shaw AT, Gettinger S, Cosper AK, Akhavanfard S, Heist RS, Temel J (2011). Genotypic and histological evolution of lung cancers acquiring resistance to EGFR inhibitors. Sci Transl Med.

[8] Bean J, Brennan C, Shih JY, Riely G, Viale A, Wang L, Chitale D, Motoi N, Szoke J, Broderick S, Balak M, Chang WC, Yu CJ (2007). MET amplification occurs with or without T790M mutations in EGFR mutant lung tumors with acquired resistance to gefitinib or erlotinib. Proc Natl Acad Sci U S A.

[9] Engelman JA, Zejnullahu K, Mitsudomi T, Song Y, Hyland C, Park JO, Lindeman N, Gale CM, Zhao X, Christensen J, Kosaka T, Holmes AJ, Rogers AM (2007). MET amplification leads to gefitinib resistance in lung cancer by activating ERBB3 signaling. Science.

[10] Zhang Z, Lee JC, Lin L, Olivas V, Au V, LaFramboise T, Abdel-Rahman M, Wang X, Levine AD, Rho JK, Choi YJ, Choi CM, Kim SW (2012). Activation of the AXL kinase causes resistance to EGFR-targeted therapy in lung cancer. Nat Genet.

[11] Yu HA, Arcila ME, Rekhtman N, Sima CS, Zakowski MF, Pao W, Kris MG, Miller VA, Ladanyi M, Riely GJ (2013). Analysis of tumor specimens at the time of acquired resistance to EGFR-TKI therapy in 155 patients with EGFR-mutant lung cancers. Clin Cancer Res.

[12] Kobayashi S, Boggon TJ, Dayaram T, Janne PA, Kocher O, Meyerson M, Johnson BE, Eck MJ, Tenen DG, Halmos B (2005). EGFR mutation and resistance of non-small-cell lung cancer to gefitinib. N Engl J Med.

[13] Fan W, Tang Z, Yin L, Morrison B, Hafez-Khayyata S, Fu P, Huang H, Bagai R, Jiang S, Kresak A, Howell S, Vasanji A, Flask CA (2011). MET-independent lung cancer cells evading EGFR kinase inhibitors are therapeutically susceptible to BH3 mimetic agents. Cancer Res.

[14] Ward PS, Thompson CB (2012). Metabolic reprogramming: a cancer hallmark even warburg did not anticipate. Cancer Cell.

[15] Hanahan D, Weinberg RA (2011). Hallmarks of cancer: the next generation. Cell.

[16] Bachman KE, Park BH (2005). Duel nature of TGF-beta signaling: tumor suppressor *vs*. tumor promoter. Curr Opin Oncol.

[17] Inman GJ (2011). Switching TGFbeta from a tumor suppressor to a tumor promoter. Curr Opin Genet Dev.

[18] Singh A, Settleman J (2010). EMT, cancer stem cells and drug resistance: an emerging axis of evil in the war on cancer. Oncogene.

[19] Oshimori N, Fuchs E (2012). The harmonies played by TGF-beta in stem cell biology. Cell Stem Cell.

[20] Billard C (2013). BH3 mimetics: status of the field and new developments. Mol Cancer Ther.

[21] Tse C, Shoemaker AR, Adickes J, Anderson MG, Chen J, Jin S, Johnson EF, Marsh KC, Mitten MJ, Nimmer P, Roberts L, Tahir SK, Xiao Y (2008). ABT-263: a potent and orally bioavailable Bcl-2 family inhibitor. Cancer Res.

[22] Souers AJ, Leverson JD, Boghaert ER, Ackler SL, Catron ND, Chen J, Dayton BD, Ding H, Enschede SH, Fairbrother WJ, Huang DC, Hymowitz SG, Jin S (2013). ABT-199, a potent and selective BCL-2 inhibitor, achieves antitumor activity while sparing platelets. Nat Med.

[23] Davids MS, Letai A (2013). ABT-199: taking dead aim at BCL-2. Cancer Cell.

[24] Paik PK, Rudin CM, Pietanza MC, Brown A, Rizvi NA, Takebe N, Travis W, James L, Ginsberg MS, Juergens R, Markus S, Tyson L, Subzwari S (2011). A phase II study of obatoclax mesylate, a Bcl-2 antagonist, plus topotecan in relapsed small cell lung cancer. Lung Cancer.

[25] Urtishak KA, Edwards AY, Wang LS, Hudome A, Robinson BW, Barrett JS, Cao K, Cory L, Moore JS, Bantly AD, Yu QC, Chen IM, Atlas SR (2013). Potent obatoclax cytotoxicity and activation of triple death mode killing across infant acute lymphoblastic leukemia. Blood.

[26] Sharma SV, Lee DY, Li B, Quinlan MP, Takahashi F, Maheswaran S, McDermott U, Azizian N, Zou L, Fischbach MA, Wong KK, Brandstetter K, Wittner B A chromatin-mediated reversible drug-tolerant state in cancer cell subpopulations. Cell.

[27] Workman P, Travers J (464). Cancer: drug-tolerant insurgents. Nature.

[28] Locasale JW, Cantley LC (2010). Altered metabolism in cancer. BMC Biol.

[29] Locasale JW, Cantley LC (2011). Metabolic flux and the regulation of mammalian cell growth. Cell Metab.

[30] Locasale JW, Cantley LC, Vander Heiden MG (2009). Cancer's insatiable appetite. Nat Biotechnol.

[31] Locasale JW, Vander Heiden MG, Cantley LC (2010). Rewiring of glycolysis in cancer cell metabolism. Cell Cycle.

[32] Davids MS, Letai A (2012). Targeting the B-cell lymphoma/leukemia 2 family in cancer. J Clin Oncol.

[33] Ni Chonghaile T, Sarosiek KA, Vo TT, Ryan JA, Tammareddi A, Moore Vdel G, Deng J, Anderson KC, Richardson P, Tai YT, Mitsiades CS, Matulonis UA, Drapkin R (2011). Pretreatment mitochondrial priming correlates with clinical response to cytotoxic chemotherapy. Science.

[34] Dannenberg JH, Berns A Drugging drug resistance. Cell.

[35] Zeitlin BD, Zeitlin IJ, Nor JE (2008). Expanding circle of inhibition: small-molecule inhibitors of Bcl-2 as anticancer cell and antiangiogenic agents. J Clin Oncol.

[36] Kutuk O, Letai A (2008). Alteration of the mitochondrial apoptotic pathway is key to acquired paclitaxel resistance and can be reversed by ABT-737. Cancer Res.

[37] Gong Y, Somwar R, Politi K, Balak M, Chmielecki J, Jiang X, Pao W (2007). Induction of BIM is essential for apoptosis triggered by EGFR kinase inhibitors in mutant EGFR-dependent lung adenocarcinomas. PLoS Med.

[38] DeBerardinis RJ, Cheng T (2010). Q's next: the diverse functions of glutamine in metabolism, cell biology and cancer. Oncogene.

[39] Yuneva M, Zamboni N, Oefner P, Sachidanandam R, Lazebnik Y (2007). Deficiency in glutamine but not glucose induces MYC-dependent apoptosis in human cells. J Cell Biol.

[40] Massague J (2012). TGFbeta signalling in context. Nat Rev Mol Cell Biol.

[41] Massague J (2008). TGFbeta in Cancer. Cell.

[42] Pickup M, Novitskiy S, Moses HL (2013). The roles of TGFbeta in the tumour microenvironment. Nat Rev Cancer.

[43] Rosell R, Bivona TG, Karachaliou N (2013). Genetics and biomarkers in personalisation of lung cancer treatment. Lancet.

[44] Huang S, Holzel M, Knijnenburg T, Schlicker A, Roepman P, McDermott U, Garnett M, Grernrum W, Sun C, Prahallad A, Groenendijk FH, Mittempergher L, Nijkamp W (2012). MED12 controls the response to multiple cancer drugs through regulation of TGF-beta receptor signaling. Cell.

[45] Rosell R (2013). Mediating resistance in oncogene-driven cancers. N Engl J Med.

[46] Cragg MS, Harris C, Strasser A, Scott CL (2009). Unleashing the power of inhibitors of oncogenic kinases through BH3 mimetics. Nat Rev Cancer.

[47] Tang Z, Jiang S, Du R, Petri ET, El-Telbany A, Chan PS, Kijima T, Dietrich S, Matsui K, Kobayashi M, Sasada S, Okamoto N, Suzuki H (2009). Disruption of the EGFR E884-R958 ion pair conserved in the human kinome differentially alters signaling and inhibitor sensitivity. Oncogene.

[48] Choong NW, Dietrich S, Seiwert TY, Tretiakova MS, Nallasura V, Davies GC, Lipkowitz S, Husain AN, Salgia R, Ma PC (2006). Gefitinib response of erlotinib-refractory lung cancer involving meninges—role of EGFR mutation. Nat Clin Pract Oncol.

[49] Tang Z, Du R, Jiang S, Wu C, Barkauskas DS, Richey J, Molter J, Lam M, Flask C, Gerson S, Dowlati A, Liu L, Lee Z (2008). Dual MET-EGFR combinatorial inhibition against T790M-EGFR-mediated erlotinib-resistant lung cancer. Br J Cancer.

