# Supplementary material for: Transcriptomic-metabolomic reprogramming in EGFR-mutant NSCLC early adaptive drug escape linking TGFβ2-bioenergetics-mitochondrial priming
Source: Oncotarget. 2016 Nov 11;7(50):82013–27. doi: 10.18632/oncotarget.13307 (PMC5347670; doi:10.18632/oncotarget.13307)
Supplement: Supplementary file 1 [file oncotarget-07-82013-s001.pdf]

Transcriptomic-metabolomic reprogramming in EGFR-mutant NSCLC early adaptive drug escape linking TGFβ2-bioenergetics-mitochondrial priming

Supplementary Material

Supplemental Figure 1

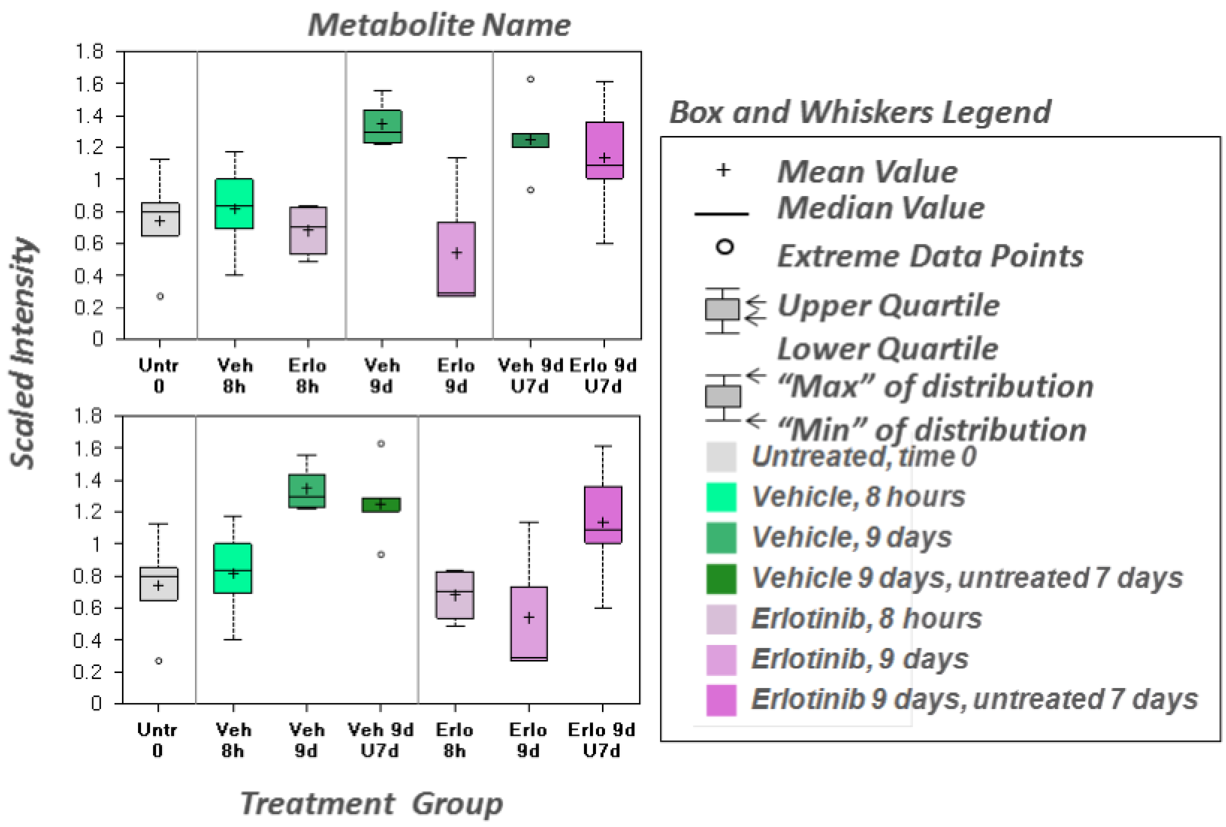

Fig. S1. An example figure to illustrate the data representation for each biochemical displayed as box plots in the metabolomics profiling study.

Supplemental Figure 2

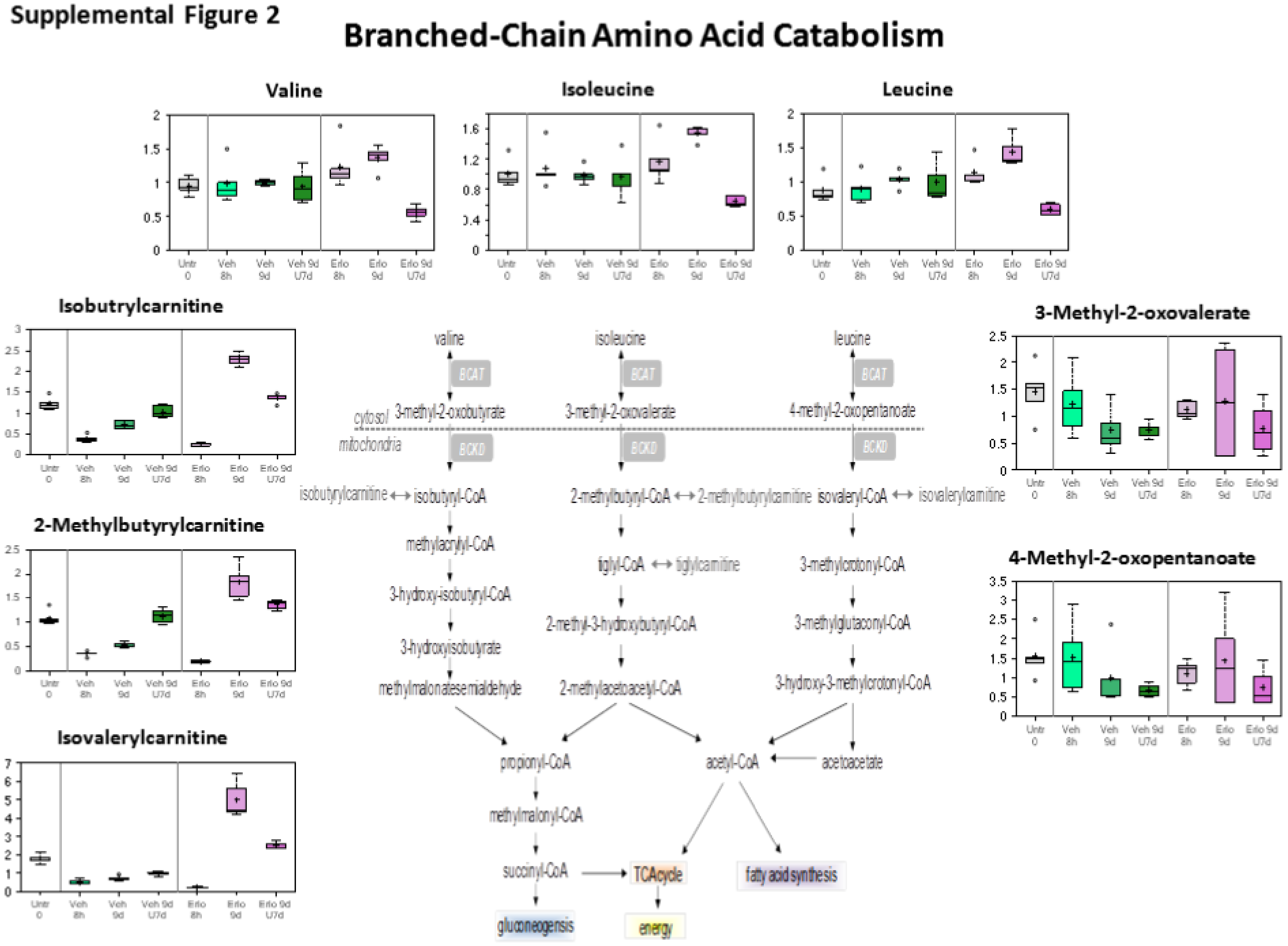

Fig. S2. Branched-chain amino acid catabolism reprogramming.

Supplemental Figure 3

Inflammation

- **Arachidonic Acid and Prostaglandins**

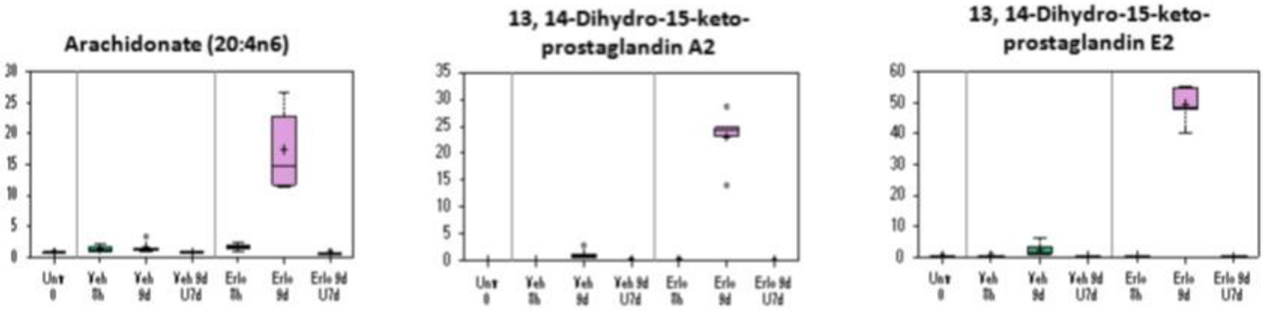

- **Endocannabinoids**

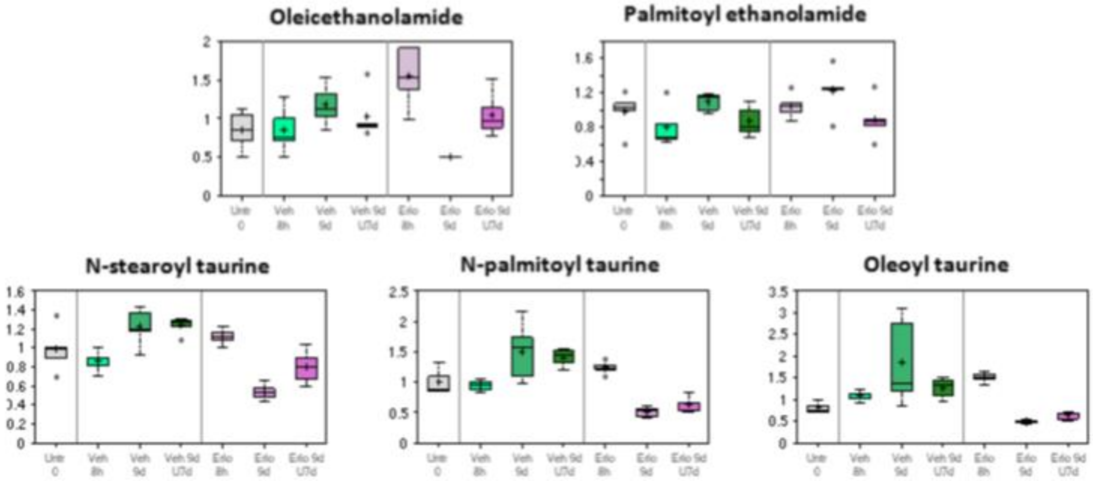

Fig. S3. Inflammation and endocannabinoids metabolism reprogramming.

Supplemental Figure 4

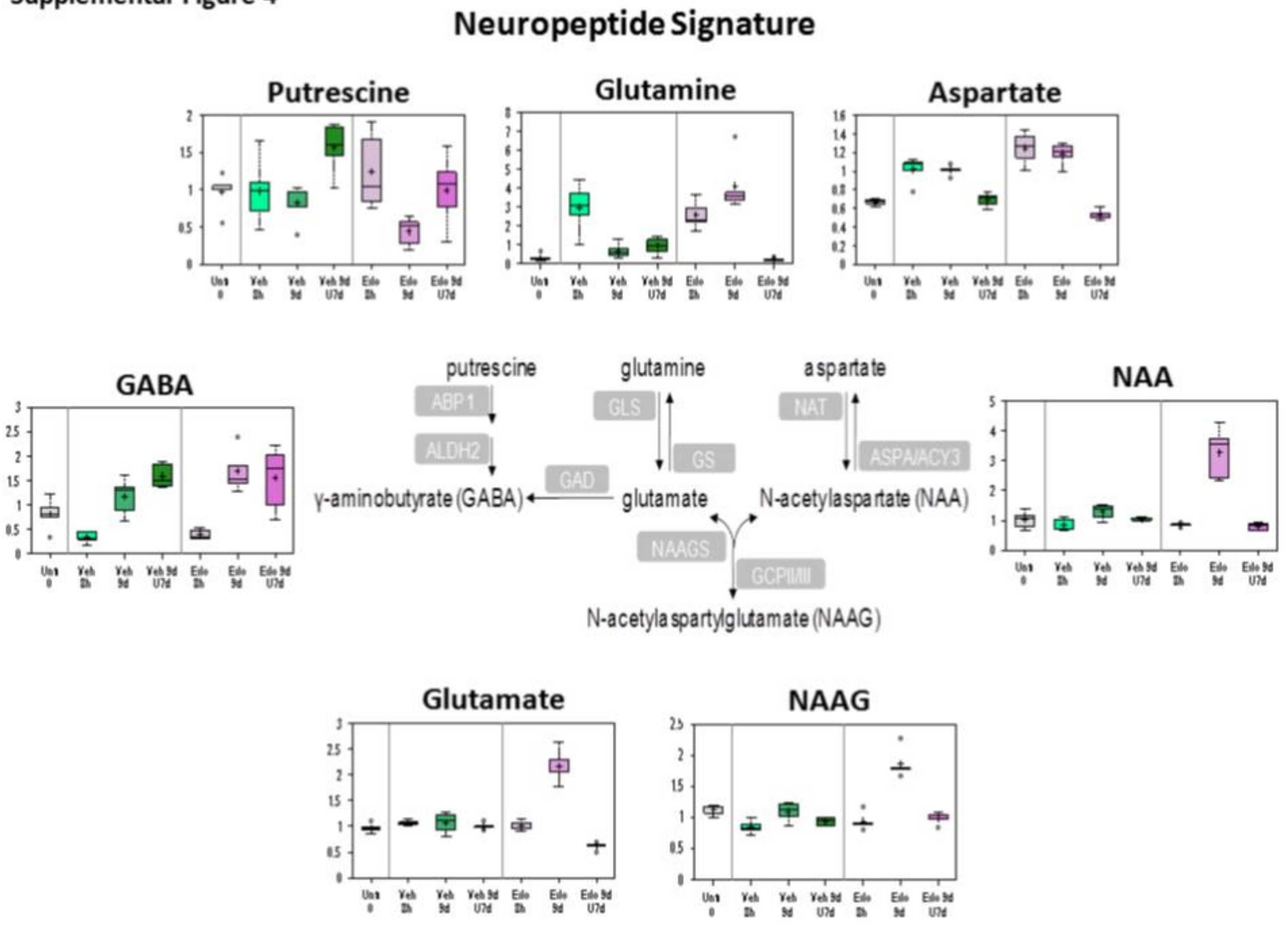

Fig. S4. Neuropeptide signature reprogramming.

Supplemental Figure 5

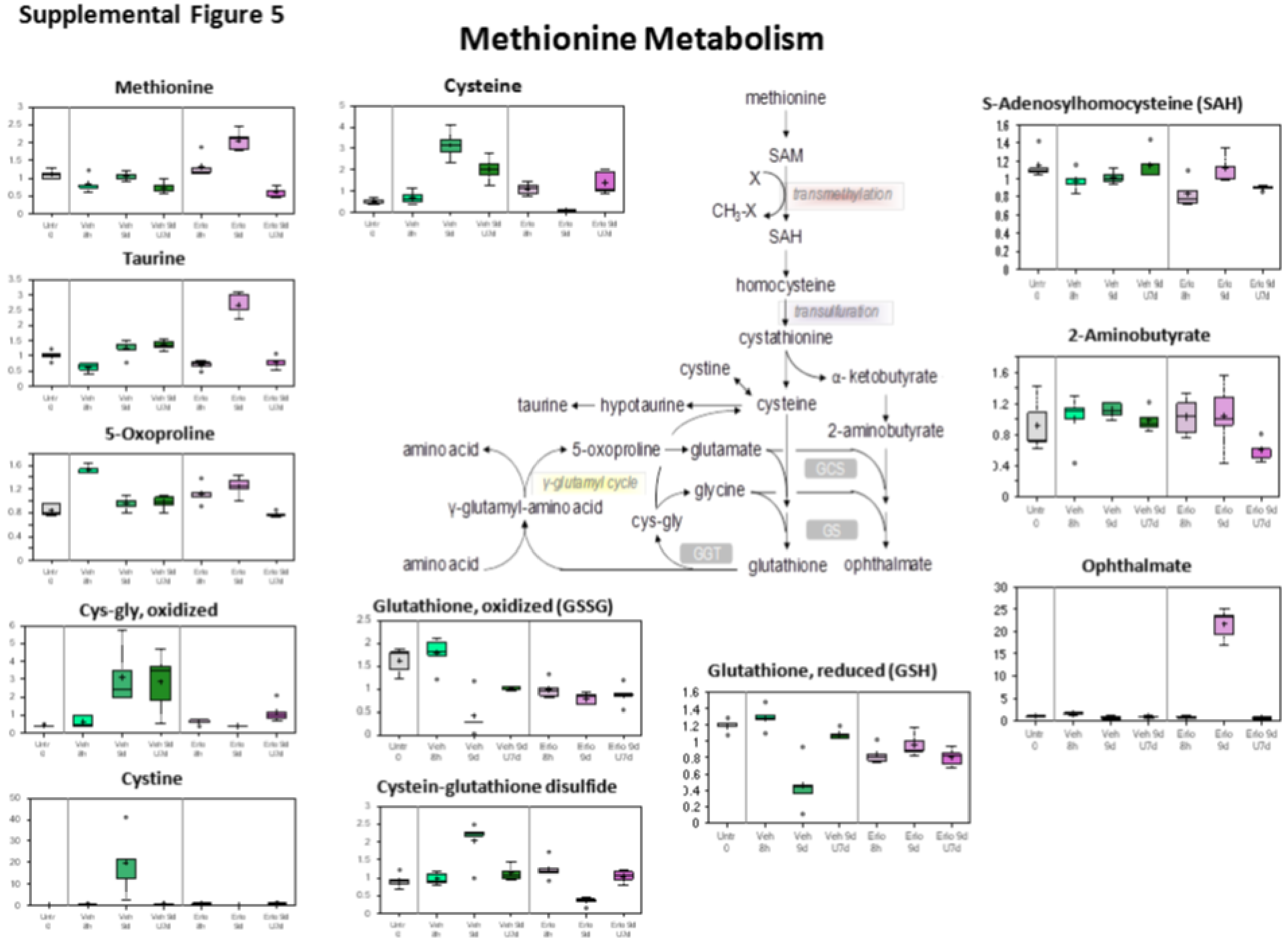

Fig. S5. Methionine metabolism and oxidative stress reprogramming.

Supplemental Figure 6

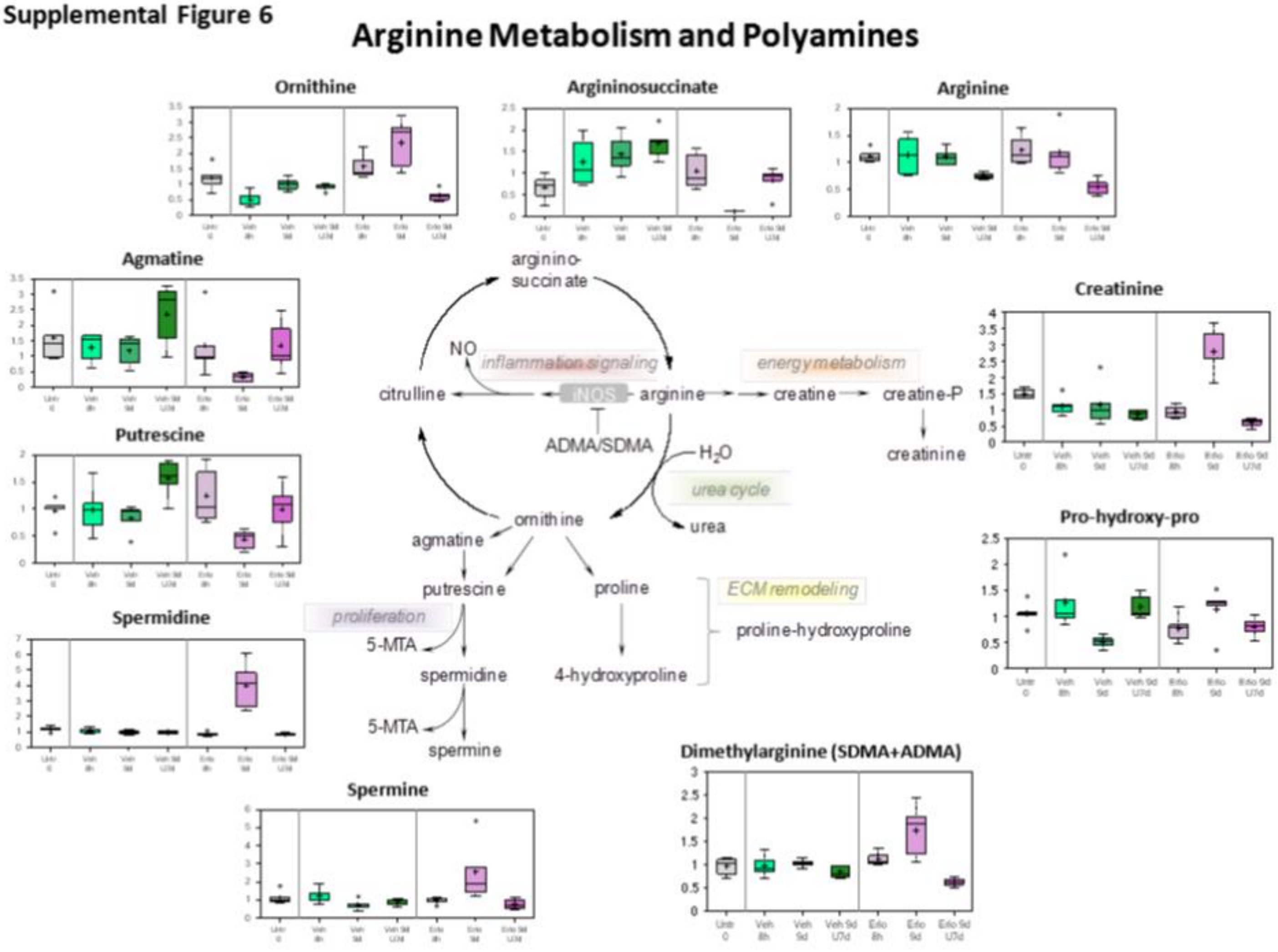

Fig. S6. Arginine and polyamine metabolism reprogramming.
