# Supplementary material for: Transcriptomic-metabolomic reprogramming in EGFR-mutant NSCLC early adaptive drug escape linking TGFβ2-bioenergetics-mitochondrial priming
Source: Oncotarget. 2016 Nov 11;7(50):82013–27. doi: 10.18632/oncotarget.13307 (PMC5347670; doi:10.18632/oncotarget.13307)
Supplement: Supplementary file 2 [file oncotarget-07-82013-s002.docx]

**Table S1.** Pathway analysis of upregulated genes in transcriptomic profiling of early adaptive drug escape against EGFR TKI in *EGFR*-mutant lung

adenocarcinoma cells – Key role of autocrine TGFβ2 Signaling.

**Supplementary Table 1**

| Name | Type | Total Entities | Expanded # of Entities | Overlap | Percent Overlap | Overlapping Entities | p-value | Data Source | # |
| --- | --- | --- | --- | --- | --- | --- | --- | --- | --- |
| cell adhesion | Group | 650 | 650 | 9 | 1 | PCDHB13,PCDHB2,FN1,CYR61,SDC4,EDIL3,NEDD9,COL12A1,FAT4 | 6.03E-07 | biological_process | 1 |
| cell proliferation | Group | 385 | 385 | 5 | 1 | CYP1A1,CYR61,TGFB2,GAS6,TACSTD2 | 0.000325 | biological_process | 9 |
| multicellular organismal development | Group | 1080 | 1080 | 5 | 0 | CLU,BMP1,HHAT,EDIL3,BICC1 | 0.025855 | biological_process | 140 |
| extracellular matrix organization | Group | 94 | 94 | 4 | 4 | FN1,CYR61,TGFB2,CCDC80 | 1.42E-05 | biological_process | 3 |
| response to hypoxia | Group | 184 | 184 | 4 | 2 | EDN1,CYP1A1,TGFB2,PDE5A | 0.000195 | biological_process | 7 |
| positive regulation of cell-substrate adhesion | Group | 26 | 26 | 3 | 11 | CYR61,EDIL3,CCDC80 | 9.00E-06 | biological_process | 2 |
| response to wounding | Group | 60 | 60 | 3 | 5 | FN1,CYP1A1,CLU | 0.000114 | biological_process | 6 |
| response to cytokine stimulus | Group | 77 | 77 | 3 | 3 | TIMP2,TGFB2,CDKN2B | 0.00024 | biological_process | 8 |
| cell cycle arrest | Group | 115 | 115 | 3 | 2 | TGFB2,CDKN2B,TP53INP1 | 0.000776 | biological_process | 13 |
| response to organic substance | Group | 123 | 123 | 3 | 2 | CYP1B1,CYP1A1,ASAH1 | 0.000943 | biological_process | 16 |
| skeletal system development | Group | 147 | 147 | 3 | 2 | EDN1,TGFB2,COL12A1 | 0.001575 | biological_process | 30 |
| homophilic cell adhesion | Group | 159 | 159 | 3 | 1 | PCDHB13,PCDHB2,FAT4 | 0.00197 | biological_process | 33 |
| anti-apoptosis | Group | 198 | 198 | 3 | 1 | FN1,CLU,PEA15 | 0.003661 | biological_process | 43 |
| response to stress | Group | 239 | 239 | 3 | 1 | CYP1A1,CLU,TP53INP1 | 0.006175 | biological_process | 55 |
| cell-cell signaling | Group | 275 | 275 | 3 | 1 | EDN1,TGFB2,SDC4 | 0.009064 | biological_process | 75 |
| response to drug | Group | 295 | 295 | 3 | 1 | TIMP2,CYP1A1,TGFB2 | 0.010959 | biological_process | 89 |
| negative regulation of cell proliferation | Group | 324 | 324 | 3 | 0 | TIMP2,TGFB2,CDKN2B | 0.014086 | biological_process | 107 |
| positive regulation of cell proliferation | Group | 332 | 332 | 3 | 0 | EDN1,TGFB2,CLU | 0.015029 | biological_process | 108 |
| dibenzo-p-dioxin metabolic process | Group | 4 | 4 | 2 | 50 | CYP1B1,CYP1A1 | 1.43E-05 | biological_process | 4 |
| toxin metabolic process | Group | 8 | 8 | 2 | 25 | CYP1B1,CYP1A1 | 6.63E-05 | biological_process | 5 |
| gut development | Group | 18 | 18 | 2 | 11 | CYP1A1,TGFB2 | 0.000359 | biological_process | 10 |
| patterning of blood vessels | Group | 24 | 24 | 2 | 8 | EDN1,CYR61 | 0.000643 | biological_process | 11 |
| cartilage condensation | Group | 24 | 24 | 2 | 8 | TGFB2,BMP1 | 0.000643 | biological_process | 12 |
| negative regulation of epithelial cell proliferation | Group | 28 | 28 | 2 | 7 | TGFB2,CDKN2B | 0.000878 | biological_process | 14 |
| neutrophil chemotaxis | Group | 28 | 28 | 2 | 7 | EDN1,TGFB2 | 0.000878 | biological_process | 15 |
| collagen fibril organization | Group | 31 | 31 | 2 | 6 | TGFB2,COL12A1 | 0.001076 | biological_process | 17 |
| calcium-dependent cell-cell adhesion | Group | 33 | 33 | 2 | 6 | PCDHB13,PCDHB2 | 0.00122 | biological_process | 18 |
| positive regulation of cell growth | Group | 39 | 39 | 2 | 5 | HBEGF,TGFB2 | 0.001702 | biological_process | 31 |
| synaptogenesis | Group | 41 | 41 | 2 | 4 | PCDHB13,PCDHB2 | 0.00188 | biological_process | 32 |
| positive regulation of smooth muscle cell proliferation | Group | 43 | 43 | 2 | 4 | HBEGF,EDN1 | 0.002066 | biological_process | 34 |
| cartilage development | Group | 54 | 54 | 2 | 3 | EDN1,BMP1 | 0.003238 | biological_process | 42 |
| positive regulation of cell migration | Group | 66 | 66 | 2 | 3 | CYR61,TGFB2 | 0.004797 | biological_process | 52 |
| wound healing | Group | 69 | 69 | 2 | 2 | FN1,TGFB2 | 0.005231 | biological_process | 53 |
| regulation of growth | Group | 71 | 71 | 2 | 2 | GAS6,NEDD9 | 0.00553 | biological_process | 54 |
| cell death | Group | 82 | 82 | 2 | 2 | TGFB2,CLU | 0.00731 | biological_process | 66 |
| regulation of cell growth | Group | 83 | 83 | 2 | 2 | CYR61,TGFB2 | 0.007483 | biological_process | 67 |
| ossification | Group | 101 | 101 | 2 | 1 | FN1,BMP1 | 0.01091 | biological_process | 87 |
| aging | Group | 101 | 101 | 2 | 1 | TIMP2,TGFB2 | 0.01091 | biological_process | 88 |
| cytoskeleton organization | Group | 102 | 102 | 2 | 1 | NEDD9,PTPN21 | 0.011117 | biological_process | 90 |
| lung development | Group | 106 | 106 | 2 | 1 | TGFB2,ASAH1 | 0.011963 | biological_process | 91 |
| response to organic cyclic substance | Group | 142 | 142 | 2 | 1 | CYP1A1,CDKN2B | 0.020773 | biological_process | 126 |
| angiogenesis | Group | 145 | 145 | 2 | 1 | HBEGF,TGFB2 | 0.0216 | biological_process | 127 |
| heart development | Group | 176 | 176 | 2 | 1 | EDN1,TGFB2 | 0.03091 | biological_process | 155 |
| regulation of apoptosis | Group | 177 | 177 | 2 | 1 | TGFB2,PEA15 | 0.031233 | biological_process | 156 |
| induction of apoptosis | Group | 207 | 207 | 2 | 0 | TGFB2,TP53INP1 | 0.04152 | biological_process | 179 |
| visual perception | Group | 213 | 213 | 2 | 0 | CYP1B1,TACSTD2 | 0.043712 | biological_process | 183 |
| synaptic transmission | Group | 214 | 214 | 2 | 0 | PCDHB13,PCDHB2 | 0.044081 | biological_process | 184 |
