# Supplementary material for: Transcriptomic-metabolomic reprogramming in EGFR-mutant NSCLC early adaptive drug escape linking TGFβ2-bioenergetics-mitochondrial priming
Source: Oncotarget. 2016 Nov 11;7(50):82013–27. doi: 10.18632/oncotarget.13307 (PMC5347670; doi:10.18632/oncotarget.13307)
Supplement: Supplementary file 3 [file oncotarget-07-82013-s003.docx]

**Supplementary Table 2**

**Table S2.** Pathway analysis of downregulated genes in transcriptomic profiling of early adaptive drug escape against EGFR TKI in *EGFR*-mutant

lung adenocarcinoma cells.

| Name | Type | Total Entities | Expanded # of Entities | Overlap | Percent Overlap | Overlapping Entities | p-value | Data Source | # |
| --- | --- | --- | --- | --- | --- | --- | --- | --- | --- |
| cell cycle | Group | 539 | 539 | 75 | 13 | KNTC1,FAM83D,CHEK2,CDK2,MKI67,BIRC5,CCNB1,MAD2L1,AURKA,CDC20,CKS1B,PLK1,GSG2,CENPE,AURKB,KIFC1,BRCA1,BUB1B,KIF23,FBXO5,MDC1,NDC80,CHEK1,MCM3,RACGAP1,GMNN,PTTG1,FANCD2,FANCI,CDC45L,UBE2C,MCM2,NUSAP1,SUV39H1,SMC4,MCM7,LIG1,CDC7,CDKN3,ANLN,NUF2,CDT1,MCM6,CLSPN,KIF11,KIF20B,CHAF1B,SMC2,PRC1,CIT,SGOL1,CDCA3,NCAPD2,OIP5,SPC25,DSCC1,CEP55,C18orf24,TIMELESS,CKAP5,CCNF,CDCA5,E2F8,MTBP,CDCA2,ZWILCH,NCAPG2,SASS6,ASPM,SGOL2,C13orf34,LRRCC1,NCAPH,LIN9,RBL1 | 1.32E-75 | biological_process | 1 |
| cell division | Group | 266 | 266 | 55 | 20 | KNTC1,FAM83D,CDK2,BIRC5,CCNB1,MAD2L1,CDC20,CKS1B,PLK1,CENPE,AURKB,KIFC1,BUB1B,KIF23,FBXO5,CENPF,NDC80,RACGAP1,PTTG1,CDC45L,UBE2C,NUSAP1,SMC4,LIG1,CDC7,ANLN,NUF2,MCM5,KIF11,CDCA7,KIF20B,CENPH,SMC2,PRC1,CIT,SGOL1,CDCA3,NCAPD2,OIP5,SPC25,CEP55,C18orf24,TIMELESS,CKAP5,CENPO,CCNF,CDCA5,CDCA2,ZWILCH,NCAPG2,ASPM,SGOL2,C13orf34,LRRCC1,NCAPH | 1.34E-64 | biological_process | 2 |
| mitosis | Group | 213 | 213 | 50 | 23 | KNTC1,FAM83D,CDK2,BIRC5,CCNB1,MAD2L1,AURKA,CDC20,PLK1,CENPE,AURKB,TPX2,KIFC1,BUB1B,KIF23,FBXO5,CENPF,NDC80,PTTG1,UBE2C,NUSAP1,SMC4,KIF22,ANLN,NUF2,KIF11,KIF20B,CENPH,SMC2,YEATS4,CIT,SGOL1,CDCA3,NCAPD2,OIP5,SPC25,CEP55,C18orf24,TIMELESS,CKAP5,CCNF,CDCA5,CDCA2,ZWILCH,NCAPG2,ASPM,C13orf34,LRRCC1,NCAPH,KIF15 | 1.29E-61 | biological_process | 3 |
| DNA replication | Group | 168 | 168 | 37 | 22 | GINS1,POLD3,GINS3,BRCA1,MCM10,CHEK1,MCM3,CDC45L,MCM2,RRM1,MCM7,TOP2A,LIG1,POLE,BLM,CDC7,CDT1,MCM4,MCM5,MCM6,CLSPN,CHAF1B,CCDC88A,ORC1L,RFC4,DSCC1,RFC5,RMI1,DTL,GINS4,GINS2,POLD1,RFC2,PRIM2,POLE2,POLQ,LIN9 | 2.50E-44 | biological_process | 4 |
| response to DNA damage stimulus | Group | 236 | 236 | 29 | 12 | NEIL3,ATAD5,CHEK2,RAD51,RAD54L,BARD1,BRCA2,BRCA1,MDC1,CHEK1,FANCG,PTTG1,FANCD2,FANCI,FANCM,MCM7,TOP2A,LIG1,POLE,BLM,CLSPN,CHAF1B,EME1,RAD54B,TIMELESS,DTL,RAD51AP1,FANCB,POLQ | 4.35E-27 | biological_process | 5 |
| DNA repair | Group | 251 | 251 | 28 | 11 | NEIL3,RAD51,RAD54L,BRCA2,BRCA1,MDC1,CHEK1,FANCG,PTTG1,FANCD2,FANCI,FANCM,TOP2A,LIG1,POLE,BLM,KIF22,CLSPN,CHAF1B,RUVBL2,RFC4,EME1,RFC5,RAD54B,RAD51AP1,FANCB,POLE2,POLQ | 5.41E-25 | biological_process | 6 |
| transcription | Group | 2246 | 2246 | 28 | 1 | FOXM1,MYBL2,ATAD2,MCM3,HLTF,EZH2,WHSC1,MCM2,SUV39H1,MCM7,CDCA7L,MCM4,MCM5,MCM6,CDCA7,CHAF1B,YEATS4,ASF1B,RUVBL2,RAD54B,TIMELESS,ZNF367,E2F8,PRIM2,MLF1IP,ZNF165,PHF19,RBL1 | 0.003609 | biological_process | 74 |
| regulation of transcription, DNA-dependent | Group | 2501 | 2501 | 28 | 1 | ZNF695,FOXM1,MYBL2,BRCA2,ATAD2,MCM3,EZH2,WHSC1,MCM2,SUV39H1,MCM7,CDCA7L,MCM4,MCM5,MCM6,CDCA7,CHAF1B,YEATS4,ASF1B,RUVBL2,RAD54B,TIMELESS,ZNF367,E2F8,MLF1IP,ZNF165,PHF19,RBL1 | 0.014649 | biological_process | 133 |
| protein amino acid phosphorylation | Group | 739 | 739 | 16 | 2 | MASTL,CHEK2,CDK2,CCNB1,AURKA,PLK1,GSG2,AURKB,BUB1B,CHEK1,TTK,PLK4,CDC7,MELK,VRK1,CIT | 0.00011 | biological_process | 42 |
| cell proliferation | Group | 385 | 385 | 13 | 3 | MKI67,SKP2,BRCA2,CKS1B,PLK1,TPX2,BUB1B,CENPF,MCM7,STIL,TRAIP,E2F8,KIF15 | 5.48E-06 | biological_process | 27 |
| nucleosome assembly | Group | 139 | 139 | 12 | 8 | HIST2H2AA3,HIST1H2AB,HIST1H2BM,HIST1H2BC,HIST1H1B,CENPA,MCM2,ASF1B,HIST2H2AC,HIST1H1E,HIST1H2BJ,HIST1H4D | 4.72E-10 | biological_process | 12 |
| modification-dependent protein catabolic process | Group | 501 | 501 | 12 | 2 | SKP2,CDC20,FBXO5,HERC5,UBE2C,CDCA3,DTL,MTBP,PPIL5,UBE2S,UBE2T,STAMBPL1 | 0.000329 | biological_process | 53 |
| microtubule-based movement | Group | 120 | 120 | 10 | 8 | TUBB2C,CENPE,KIFC1,KIF23,KIF22,KIF11,KIF20B,KIF14,KIF18A,KIF15 | 1.93E-08 | biological_process | 16 |
| chromosome segregation | Group | 39 | 39 | 9 | 23 | ESPL1,BRCA1,PTTG1,TOP2A,NUF2,CENPH,SGOL1,CENPO,SGOL2 | 8.39E-12 | biological_process | 9 |
| DNA recombination | Group | 69 | 69 | 9 | 13 | RAD54L,BRCA2,LIG1,BLM,MND1,RUVBL2,EME1,PSMC3IP,RAD51AP1 | 1.86E-09 | biological_process | 14 |
| chromatin modification | Group | 186 | 186 | 9 | 4 | GSG2,HLTF,EZH2,WHSC1,SUV39H1,YEATS4,ASF1B,RUVBL2,RBL1 | 9.52E-06 | biological_process | 29 |
| nucleotide-excision repair, DNA gap filling | Group | 17 | 17 | 8 | 47 | POLD3,LIG1,POLE,RFC4,RFC5,POLD1,RFC2,POLE2 | 1.57E-13 | biological_process | 7 |
| DNA replication initiation | Group | 21 | 21 | 8 | 38 | MCM3,CDC45L,MCM2,MCM7,MCM4,MCM5,MCM6,ORC1L | 1.28E-12 | biological_process | 8 |
| response to drug | Group | 295 | 295 | 8 | 2 | CDK2,ADFP,RAD51,RAD54L,CENPF,GGH,ENO2,RAD54B | 0.001498 | biological_process | 64 |
| double-strand break repair via homologous recombination | Group | 16 | 16 | 7 | 43 | RAD51,RAD54L,BRCA2,BRCA1,BLM,RAD54B,RAD51AP1 | 1.05E-11 | biological_process | 10 |
| mitotic chromosome condensation | Group | 21 | 21 | 7 | 33 | NUSAP1,SMC4,SMC2,NCAPD2,CDCA5,NCAPG2,NCAPH | 1.04E-10 | biological_process | 11 |
| cytokinesis | Group | 39 | 39 | 7 | 17 | BIRC5,AURKB,ESPL1,RACGAP1,ANLN,PRC1,CIT | 1.23E-08 | biological_process | 15 |
| mitotic sister chromatid segregation | Group | 16 | 16 | 6 | 37 | MAD2L1,ESPL1,KIFC1,NDC80,NUSAP1,CIT | 1.04E-09 | biological_process | 13 |
| chromosome organization | Group | 35 | 35 | 6 | 17 | RAD54L,BRCA2,PTTG1,SMC4,BLM,SMC2 | 1.88E-07 | biological_process | 19 |
| phosphoinositide-mediated signaling | Group | 47 | 47 | 6 | 12 | AURKA,BUB1B,NDC80,UBE2C,TOP2A,RFC4 | 1.15E-06 | biological_process | 23 |
| DNA metabolic process | Group | 59 | 59 | 6 | 10 | RAD51,PTTG1,FANCM,TOP2A,KPNA2,EME1 | 4.50E-06 | biological_process | 26 |
| positive regulation of cell proliferation | Group | 332 | 332 | 6 | 1 | CDK2,FOXM1,CENPF,TTK,CDC7,CDCA7L | 0.03509 | biological_process | 170 |
| DNA unwinding during replication | Group | 14 | 14 | 5 | 35 | RAD51,MCM2,MCM7,MCM4,MCM6 | 3.68E-08 | biological_process | 17 |
| spindle organization | Group | 16 | 16 | 5 | 31 | AURKA,RANBP1,BUB1B,NDC80,UBE2C | 7.94E-08 | biological_process | 18 |
| G2-M transition of mitotic cell cycle | Group | 22 | 22 | 5 | 22 | CDK2,BIRC5,CCNB1,CHEK1,CIT | 4.62E-07 | biological_process | 20 |
| regulation of cyclin-dependent protein kinase activity | Group | 42 | 42 | 5 | 11 | CKS1B,CHEK1,HERC5,BLM,CDKN3 | 1.33E-05 | biological_process | 31 |
| G1-S transition of mitotic cell cycle | Group | 57 | 57 | 5 | 8 | SKP2,POLE,CDC7,CDKN3,CDCA5 | 5.97E-05 | biological_process | 39 |
| negative regulation of ubiquitin-protein ligase activity during mitotic cell cycle | Group | 64 | 64 | 5 | 7 | MAD2L1,CDC20,BUB1B,FBXO5,UBE2C | 0.000104 | biological_process | 40 |
| anaphase-promoting complex-dependent proteasomal ubiquitin-dependent protein catabolic process | Group | 64 | 64 | 5 | 7 | CCNB1,MAD2L1,CDC20,BUB1B,UBE2C | 0.000104 | biological_process | 41 |
| positive regulation of ubiquitin-protein ligase activity during mitotic cell cycle | Group | 67 | 67 | 5 | 7 | CCNB1,CDC20,PLK1,FBXO5,UBE2C | 0.00013 | biological_process | 43 |
| meiosis | Group | 76 | 76 | 5 | 6 | MKI67,RAD51,RAD54L,MND1,PSMC3IP | 0.000236 | biological_process | 49 |
| cell cycle arrest | Group | 115 | 115 | 5 | 4 | GAS2L3,BARD1,CDKN3,KIF20B,MTBP | 0.001559 | biological_process | 65 |
| regulation of cell proliferation | Group | 135 | 135 | 5 | 3 | FOXM1,BRCA1,CDCA7,CCDC88A,TIMELESS | 0.003134 | biological_process | 72 |
| negative regulation of cell cycle | Group | 156 | 156 | 5 | 3 | BRCA1,BUB1B,GMNN,LIN9,RBL1 | 0.005775 | biological_process | 79 |
| mitotic cell cycle spindle assembly checkpoint | Group | 11 | 11 | 4 | 36 | MAD2L1,CENPE,CENPF,TTK | 8.62E-07 | biological_process | 21 |
| centrosome duplication | Group | 11 | 11 | 4 | 36 | BRCA2,BRCA1,Cp110,SASS6 | 8.62E-07 | biological_process | 22 |
| mitotic cell cycle checkpoint | Group | 15 | 15 | 4 | 26 | KNTC1,MAD2L1,BUB1B,ZWILCH | 3.49E-06 | biological_process | 24 |
| oocyte maturation | Group | 15 | 15 | 4 | 26 | CCNB1,BRCA2,FBXO5,TRIP13 | 3.49E-06 | biological_process | 25 |
| DNA damage response, signal transduction resulting in induction of apoptosis | Group | 21 | 21 | 4 | 19 | CHEK2,BRCA2,BRCA1,UACA | 1.48E-05 | biological_process | 32 |
| reciprocal meiotic recombination | Group | 26 | 26 | 4 | 15 | RAD51,CHEK1,RAD54B,TRIP13 | 3.59E-05 | biological_process | 34 |
| mitotic metaphase | Group | 6 | 6 | 3 | 50 | CENPE,FBXO5,NDC80 | 7.47E-06 | biological_process | 28 |
| G2 phase of mitotic cell cycle | Group | 7 | 7 | 3 | 42 | CENPF,BLM,KPNA2 | 1.30E-05 | biological_process | 30 |
| DNA replication checkpoint | Group | 9 | 9 | 3 | 33 | CDC45L,CDT1,CLSPN | 3.09E-05 | biological_process | 33 |
| inner cell mass cell proliferation | Group | 10 | 10 | 3 | 30 | GINS1,BRCA2,NCAPG2 | 4.38E-05 | biological_process | 35 |
| DNA synthesis during DNA repair | Group | 10 | 10 | 3 | 30 | POLD3,POLE,POLD1 | 4.38E-05 | biological_process | 36 |
| response to gamma radiation | Group | 23 | 23 | 3 | 13 | CHEK2,BRCA2,FANCD2 | 0.000603 | biological_process | 55 |
| double-strand break repair | Group | 30 | 30 | 3 | 10 | RAD54L,BRCA2,TRIP13 | 0.001333 | biological_process | 63 |
| gluconeogenesis | Group | 33 | 33 | 3 | 9 | PGK1,GPI,ENO2 | 0.001762 | biological_process | 66 |
| glycolysis | Group | 68 | 68 | 3 | 4 | PGK1,GPI,ENO2 | 0.013445 | biological_process | 111 |
| regulation of cell cycle | Group | 69 | 69 | 3 | 4 | SKP2,CCNB1,CIT | 0.013982 | biological_process | 112 |
| regulation of protein metabolic process | Group | 71 | 71 | 3 | 4 | UBE2C,UBE2S,UBE2T | 0.015093 | biological_process | 134 |
| response to estradiol stimulus | Group | 79 | 79 | 3 | 3 | CDK2,AURKA,ENO2 | 0.020023 | biological_process | 137 |
| post-translational protein modification | Group | 95 | 95 | 3 | 3 | UBE2C,UBE2S,UBE2T | 0.032232 | biological_process | 167 |
| spindle pole body organization | Group | 2 | 2 | 2 | 100 | KIF11,CKAP5 | 5.27E-05 | biological_process | 37 |
| mitotic spindle elongation | Group | 2 | 2 | 2 | 100 | KIF23,PRC1 | 5.27E-05 | biological_process | 38 |
| kinetochore assembly | Group | 3 | 3 | 2 | 66 | CENPE,CENPF | 0.000157 | biological_process | 44 |
| positive regulation of mitotic metaphase-anaphase transition | Group | 3 | 3 | 2 | 66 | CENPE,ESPL1 | 0.000157 | biological_process | 45 |
| homologous chromosome segregation | Group | 3 | 3 | 2 | 66 | ESPL1,PTTG1 | 0.000157 | biological_process | 46 |
| establishment of mitotic spindle localization | Group | 3 | 3 | 2 | 66 | ESPL1,NUSAP1 | 0.000157 | biological_process | 47 |
| mitotic metaphase plate congression | Group | 3 | 3 | 2 | 66 | CENPE,CDCA5 | 0.000157 | biological_process | 48 |
| replication fork protection | Group | 4 | 4 | 2 | 50 | BRCA2,BLM | 0.000313 | biological_process | 50 |
| attachment of spindle microtubules to kinetochore | Group | 4 | 4 | 2 | 50 | CENPE,NUF2 | 0.000313 | biological_process | 51 |
| purine ribonucleoside salvage | Group | 4 | 4 | 2 | 50 | HPRT1,PRTFDC1 | 0.000313 | biological_process | 52 |
| positive regulation of exit from mitosis | Group | 5 | 5 | 2 | 40 | BIRC5,UBE2C | 0.000519 | biological_process | 54 |
| regulation of S phase | Group | 7 | 7 | 2 | 28 | CDC7,TIMELESS | 0.00108 | biological_process | 56 |
| regulation of S phase of mitotic cell cycle | Group | 7 | 7 | 2 | 28 | BRCA2,CDT1 | 0.00108 | biological_process | 57 |
| traversing start control point of mitotic cell cycle | Group | 7 | 7 | 2 | 28 | CDK2,MTBP | 0.00108 | biological_process | 58 |
| DNA damage response, signal transduction by p53 class mediator resulting in transcription of p21 class mediator | Group | 7 | 7 | 2 | 28 | BRCA2,BRCA1 | 0.00108 | biological_process | 59 |
| mitotic metaphase-anaphase transition | Group | 7 | 7 | 2 | 28 | BUB1B,CIT | 0.00108 | biological_process | 60 |
| glycine biosynthetic process | Group | 7 | 7 | 2 | 28 | DHFR,SHMT1 | 0.00108 | biological_process | 61 |
| mitotic recombination | Group | 7 | 7 | 2 | 28 | RAD51,RAD54B | 0.00108 | biological_process | 62 |
| spindle assembly | Group | 9 | 9 | 2 | 22 | TPX2,FBXO5 | 0.001833 | biological_process | 67 |
| regulation of exit from mitosis | Group | 9 | 9 | 2 | 22 | KNTC1,ANLN | 0.001833 | biological_process | 68 |
| M phase of mitotic cell cycle | Group | 10 | 10 | 2 | 20 | CENPF,MPHOSPH9 | 0.002281 | biological_process | 69 |
| meiotic chromosome segregation | Group | 10 | 10 | 2 | 20 | SGOL1,SGOL2 | 0.002281 | biological_process | 70 |
| male meiosis I | Group | 11 | 11 | 2 | 18 | BRCA2,TRIP13 | 0.002774 | biological_process | 71 |
| chordate embryonic development | Group | 12 | 12 | 2 | 16 | BRCA2,BRCA1 | 0.003313 | biological_process | 73 |
| chromosome condensation | Group | 13 | 13 | 2 | 15 | TOP2A,NCAPD2 | 0.003897 | biological_process | 75 |
| positive regulation of mitotic cell cycle | Group | 14 | 14 | 2 | 14 | BIRC5,BRCA2 | 0.004524 | biological_process | 76 |
| regulation of DNA replication | Group | 14 | 14 | 2 | 14 | CDK2,CCDC88A | 0.004524 | biological_process | 77 |
| mitotic spindle organization | Group | 15 | 15 | 2 | 13 | TTK,KIF11 | 0.005195 | biological_process | 78 |
| mitotic cell cycle | Group | 17 | 17 | 2 | 11 | AURKA,NCAPH | 0.006665 | biological_process | 80 |
| regulation of mitosis | Group | 18 | 18 | 2 | 11 | CENPE,KIF20B | 0.007463 | biological_process | 105 |
| DNA damage checkpoint | Group | 21 | 21 | 2 | 9 | CHEK2,CHEK1 | 0.010098 | biological_process | 106 |
| response to X-ray | Group | 21 | 21 | 2 | 9 | BRCA2,BLM | 0.010098 | biological_process | 107 |
| negative regulation of caspase activity | Group | 23 | 23 | 2 | 8 | BIRC5,GPI | 0.01205 | biological_process | 108 |
| regulation of phosphorylation | Group | 23 | 23 | 2 | 8 | BARD1,MCM7 | 0.01205 | biological_process | 109 |
| DNA-dependent DNA replication | Group | 23 | 23 | 2 | 8 | POLD1,POLE2 | 0.01205 | biological_process | 110 |
| response to ionizing radiation | Group | 25 | 25 | 2 | 8 | RAD54L,RAD54B | 0.014153 | biological_process | 113 |
| protein homotetramerization | Group | 27 | 27 | 2 | 7 | SHMT1,HPRT1 | 0.016403 | biological_process | 135 |
| nucleoside metabolic process | Group | 28 | 28 | 2 | 7 | HPRT1,PRTFDC1 | 0.017581 | biological_process | 136 |
| nucleotide-excision repair | Group | 32 | 32 | 2 | 6 | NEIL3,BRCA2 | 0.022636 | biological_process | 154 |
| protein oligomerization | Group | 37 | 37 | 2 | 5 | RRM1,BLM | 0.029685 | biological_process | 164 |
| multicellular organism growth | Group | 37 | 37 | 2 | 5 | BRCA2,STIL | 0.029685 | biological_process | 165 |
| response to UV | Group | 38 | 38 | 2 | 5 | UACA,POLD1 | 0.031187 | biological_process | 166 |
| negative regulation of neuron differentiation | Group | 40 | 40 | 2 | 5 | CIT,ASPM | 0.034278 | biological_process | 168 |
| one-carbon compound metabolic process | Group | 40 | 40 | 2 | 5 | DHFR,SHMT1 | 0.034278 | biological_process | 169 |
| learning or memory | Group | 44 | 44 | 2 | 4 | IL18,GPI | 0.040799 | biological_process | 185 |
| gamete generation | Group | 46 | 46 | 2 | 4 | CHEK1,FANCD2 | 0.04422 | biological_process | 193 |
