# Supplementary material for: Transcriptomic-metabolomic reprogramming in EGFR-mutant NSCLC early adaptive drug escape linking TGFβ2-bioenergetics-mitochondrial priming
Source: Oncotarget. 2016 Nov 11;7(50):82013–27. doi: 10.18632/oncotarget.13307 (PMC5347670; doi:10.18632/oncotarget.13307)
Supplement: Supplementary file 4 [file oncotarget-07-82013-s004.docx]

**Supplementary Table 3**

**Table S3.** Metabolomic profiling analysis - Metabolite summary and significantly altered biochemical. The present dataset comprises a total 356 compounds of known identity (named biochemicals).  Following log transformation, normalization to Bradford protein concentration and imputation with minimum observed values for each compound, Welch’s two-sample *t*-test was used to identify biochemicals that differed significantly between experimental groups. A summary of the numbers of biochemicals that achieved statistical significance (*p*≤0.05), as well as those approaching significance (0.05<*p*<0.10), is shown here.

| **Statistical Comparisons Welch's Two-Sample t-Test** | | | | | | | | |
| --- | --- | --- | --- | --- | --- | --- | --- | --- |
|  |  |  |  | |  | |  | |
| ***Significantly  Altered  Biochemicals*** | | Total biochemicals *p*≤0.05 | | Biochemicals (↑↓) | | Total biochemicals 0.05<*p*<0.10 | | Biochemicals (↑↓) |
| ***Erlotinib Vehicle*** | **Erlo 8h Veh 8h** | 57 | | 34\|23 | | 25 | | 17\|8 |
|  | **Erlo 9d Veh 9d** | 187 | | 126\|61 | | 24 | | 16\|8 |
|  | **Erlo 9d, U7d Veh 9d, U7d** | 142 | | 20\|122 | | 24 | | 1\|23 |
| ***Vehicle Untreated*** | **Veh 8h Untr 0** | 100 | | 37\|63 | | 21 | | 7\|14 |
|  | **Veh 9d Untr 0** | 128 | | 54\|74 | | 25 | | 10\|15 |
|  | **Veh 9d, U7d Untr 0** | 161 | | 66\|95 | | 30 | | 21\|9 |
| ***Vehicle Between Time Points*** | **Veh 9d Veh 8h** | 126 | | 78\|48 | | 38 | | 16\|22 |
|  | **Veh 9d, U7d Veh 8h** | 169 | | 89\|80 | | 30 | | 13\|17 |
|  | **Veh 9d, U7d Veh 9d** | 116 | | 53\|63 | | 32 | | 18\|14 |
| ***Erlotinib Untreated*** | **Erlo 8h Untr 0** | 108 | | 44\|64 | | 31 | | 12\|19 |
|  | **Erlo 9d Untr 0** | 189 | | 128\|61 | | 23 | | 14\|9 |
|  | **Erlo 9d, U7d Untr 0** | 142 | | 21\|121 | | 33 | | 8\|25 |
| ***Erlotinib Between Time Points*** | **Erlo 9d Erlo 8h** | 180 | | 131\|49 | | 26 | | 10\|16 |
|  | **Erlo 9d, U7d Erlo 8h** | 165 | | 48\|117 | | 32 | | 8\|24 |
|  | **Erlo 9d, U7d Erlo 9d** | 207 | | 53\|154 | | 20 | | 8\|12 |
