# Supplementary material for: Transcriptomic-metabolomic reprogramming in EGFR-mutant NSCLC early adaptive drug escape linking TGFβ2-bioenergetics-mitochondrial priming
Source: Oncotarget. 2016 Nov 11;7(50):82013–27. doi: 10.18632/oncotarget.13307 (PMC5347670; doi:10.18632/oncotarget.13307)
Supplement: Supplementary file 5 [file oncotarget-07-82013-s005.docx]

**Table S4.** Metabolomic profiling – Lipid metabolites alterations in metabolism of HCC827 cells under erlotinib treatment.

Supplementary Table 4

|  | **BIOCHEMICAL NAME** | **Erlo 8h Veh 8h** | **Erlo 9d Veh 9d** | **Erlo 9d, U7d Veh 9d, U7d** |
| --- | --- | --- | --- | --- |
| Essential fatty acid | linoleate (18:2n6) | 0.99 | 2.00 | 0.96 |
|  | linolenate [alpha or gamma; (18:3n3 or 6)] | 1.08 | 2.22 | 1.03 |
|  | dihomo-linolenate (20:3n3 or n6) | 1.22 | **2.11** | 0.87 |
|  | docosapentaenoate (n3 DPA; 22:5n3) | 1.29 | 3.10 | 0.87 |
|  | docosahexaenoate (DHA; 22:6n3) | 1.32 | 3.94 | 0.90 |
| Medium chain fatty acid | caproate (6:0) | 1.53 | 2.22 | 1.08 |
|  | heptanoate (7:0) | 0.81 | 1.16 | 1.36 |
|  | caprylate (8:0) | 1.02 | 2.65 | 1.04 |
|  | pelargonate (9:0) | 0.99 | 2.47 | 1.02 |
|  | caprate (10:0) | 0.90 | 3.16 | 1.14 |
|  | undecanoate (11:0) | 0.93 | 3.28 | **0.71** |
|  | laurate (12:0) | 0.84 | 3.05 | 1.17 |
| Long chain fatty acid | myristate (14:0) | 0.94 | 2.45 | 1.15 |
|  | myristoleate (14:1n5) | 1.00 | 2.67 | 1.12 |
|  | pentadecanoate (15:0) | 0.86 | 3.04 | 1.06 |
|  | palmitate (16:0) | 0.84 | 2.66 | 1.00 |
|  | palmitoleate (16:1n7) | 0.95 | 2.66 | 1.08 |
|  | margarate (17:0) | 0.93 | 1.85 | 1.10 |
|  | 10-heptadecenoate (17:1n7) | 0.98 | 1.71 | **0.82** |
|  | stearate (18:0) | 0.85 | 1.85 | 1.19 |
|  | oleate (18:1n9) | 0.93 | 1.68 | **0.89** |
|  | cis-vaccenate (18:1n7) | 0.90 | 1.62 | **0.86** |
|  | nonadecanoate (19:0) | 0.96 | 0.94 | 1.04 |
|  | 10-nonadecenoate (19:1n9) | 1.08 | 0.48 | 0.75 |
|  | arachidate (20:0) | 0.98 | 1.15 | 1.08 |
|  | eicosenoate (20:1n9 or 11) | 1.01 | 0.16 | 0.53 |
|  | mead acid (20:3n9) | 0.88 | 2.38 | 0.87 |
|  | arachidonate (20:4n6) | 1.23 | 10.76 | 0.87 |
|  | docosadienoate (22:2n6) | 0.98 | 0.24 | 0.62 |
|  | docosatrienoate (22:3n3) | 1.53 | 0.26 | 1.07 |
|  | adrenate (22:4n6) | 1.21 | 6.55 | 1.20 |
